# Clonal dynamics limits detection of selection in tumour xenograft CRISPR/Cas9 screens

**DOI:** 10.1038/s41417-023-00664-5

**Published:** 2023-09-08

**Authors:** Tet Woo Lee, Francis W. Hunter, Peter Tsai, Cristin G. Print, William R. Wilson, Stephen M. F. Jamieson

**Affiliations:** 1 Auckland Cancer Society Research Centre, University of Auckland, Auckland, New Zealand; 2 Maurice Wilkins Centre for Molecular Biodiscovery, University of Auckland, Auckland, New Zealand; 3 Oncology Therapeutic Area, Janssen Research and Development, Spring House, PA USA; 4 Department of Molecular Medicine and Pathology, University of Auckland, Auckland, New Zealand; 5 Department of Pharmacology and Clinical Pharmacology, University of Auckland, Auckland, New Zealand

**Keywords:** Functional genomics, Cancer models

## Abstract

Transplantable in vivo CRISPR/Cas9 knockout screens, in which cells are edited in vitro and inoculated into mice to form tumours, allow evaluation of gene function in a cancer model that incorporates the multicellular interactions of the tumour microenvironment. To improve our understanding of the key parameters for success with this method, we investigated the choice of cell line, mouse host, tumour harvesting timepoint and guide RNA (gRNA) library size. We found that high gRNA (80–95%) representation was maintained in a HCT116 subline transduced with the GeCKOv2 whole-genome gRNA library and transplanted into NSG mice when tumours were harvested at early (14 d) but not late time points (38–43 d). The decreased representation in older tumours was accompanied by large increases in variance in gRNA read counts, with notable expansion of a small number of random clones in each sample. The variable clonal dynamics resulted in a high level of ‘noise’ that limited the detection of gRNA-based selection. Using simulated datasets derived from our experimental data, we show that considerable reductions in count variance would be achieved with smaller library sizes. Based on our findings, we suggest a pathway to rationally design adequately powered in vivo CRISPR screens for successful evaluation of gene function.

## Introduction

The molecular profiling of human tumours offers unprecedented opportunities to individualise cancer therapy [1]. Detection of oncogenic mutations in patient tumours allows treatment with therapies that directly target the mutation, in some instances in a tissue-agnostic approach [2]. In addition, gene expression profiles can be used to track the emergence of resistance [3] and to identify predictive biomarkers to optimally match drugs with patients [4], but there is an incomplete understanding of the genes that determine sensitivity to most anticancer agents. One approach to remedy this is through the use of functional genomics screens, which alter gene activity by knockdown (RNA or CRISPR interference) or mutation (CRISPR/Cas9) using largescale libraries, select specific phenotypes (e.g. drug sensitivity or resistance) and then identify the gene(s) responsible [5, 6].

Whole genome functional genomic screens utilising CRISPR/Cas9 technology were first applied for evaluating genes involved in drug sensitivity or resistance in cancer cell lines in 2014 [7–9]. Since that time, a large number of in vitro whole genome screens have been undertaken utilising multiple different guide RNA (gRNA) libraries to identify essential genes [10–12] and uncover novel genes implicated in tumour growth [13, 14], drug sensitivity and resistance [15–18], synthetic lethal interactions [12, 19–21] and immunotherapy response [22, 23]. However, while CRISPR/Cas9 screens provide a powerful approach for gene discovery in vitro, the complex multicellular interactions within the tumour microenvironment [24, 25] means that the genes controlling cell growth and drug sensitivity are not necessarily the same in cell culture and in tumours. To address this, tumour models are required that are suitable for in vivo functional genomics screens.

Tumour models for in vivo CRISPR screens can be generated through direct mutagenesis in vivo or through in vivo transplantation of cells mutagenised in culture. The direct approach allows spontaneous autochthonous tumour formation with preservation of the tumour microenvironment in immunocompetent mice but is associated with numerous technical challenges, restricting its use to very small gRNA libraries of a few hundred gRNAs [26, 27]. Transplantable models, conversely, are more amenable to larger gRNA libraries. Cancer cell lines transduced at scale with larger gRNA libraries (thousands of gRNAs) can be injected into mice at large cell numbers to ensure high representation of each gRNA at inoculation [28–33]. However, the majority of transplantable in vivo CRISPR screens report a dramatic loss of clonal diversity during tumour growth due to the selection and bottleneck of cellular evolution imposed by the transition from in vitro to in vivo tumour growth [28, 29, 31, 32]. This loss of clonal diversity and gRNA representation consequently limits the discovery of genes that influence therapeutic response in these in vivo CRISPR screens. Given these limitations, the optimisation of experimental parameters would be beneficial when conducting in vivo screens. To our knowledge, however, experimental data in the literature on the importance of these parameters is limited.

In this study, we investigate the importance of several experimental parameters for in vivo CRISPR screens and their impacts on clonal diversity as measured by the distribution of gRNA counts in the tumour samples. We first evaluate the tumour-initiating ability of 22 head and neck squamous cell carcinoma lines in comparison with an HCT116 subline (HCT116/54C). We construct CRISPR/Cas9 libraries of two selected cell lines with different numbers of tumour-initiating cells and compare between the cell lines and between mouse hosts when these libraries are grown as tumour xenografts. Using the most promising combination of cell line and mouse host from our initial experiments, we then conducted a larger experiment with four experimental groups. We report that the major limiting factor in detecting gRNA-driven selection is the high variance in gRNA counts between tumours, which increases with time as the tumours grow. We use our data to simulate a reduction in library sizes and explore how this alters count variance and statistical power. Our data and analyses demonstrate the key parameters that must be optimised to ensure successful in vivo CRISPR screens and suggest a pathway for the rational design of sufficiently powered screens.

## Materials and methods

### Cell culture

The UT-SCC cell lines were derived from head and neck squamous cell carcinoma tumours by Dr Reidar Grénman (University of Turku, Turku, Finland) and supplied by Prof. Bradly Wouters (University Health Network, Toronto, Canada). HCT116/54C is an HCT116 subline that originated from a mixed culture with UT-SCC-54C cells [34]. All cell lines were passaged in minimum essential media (ThermoFisher Scientific, Waltham, MA) with 10% foetal calf serum (Moregate Biotech, Hamilton, New Zealand), 4.5 mg/mL D-glucose (Sigma-Aldrich, St Louis, MO) and 20 mM HEPES (Sigma-Aldrich) for up to 12 weeks, from authenticated frozen stocks [34] at 37 °C in humidified 5% CO_2_ incubators. All cell lines were confirmed to be Mycoplasma negative by PlasmoTest (InvivoGen, San Diego, CA). Penicillin/streptomycin (ThermoFisher Scientific) was added during the expansion of cultures for inoculation into mice.

### Lentiviral packaging

lentiCas9-Blast (AddGene, Watertown, MA; #52962) was packaged in HEK293T cells using the pMD2.G (Addgene #12259) and psPAX2 (Addgene #12260) packaging plasmids transfected with Lipofectamine 2000 (ThermoFisher Scientific). The Human GeCKOv2 A and B libraries in lentiGuide-Puro were amplified as described [35], pooled at equimolar concentrations and packaged in the same way. Filtered unconcentrated lentiCas9-Blast lentivirus was used for transductions, while GeCKOv2 library lentivirus was concentrated by centrifugation at 10,000 × *g* for 5 h with a 20% sucrose cushion.

### Transductions

To produce Cas9-expressing HCT116/54C and UT-SCC-74B cells, 5 × 10^4^ cells in normal culture medium with 8 µg/ml polybrene were transduced with lentiCas9-Blast lentivirus at a multiplicity of infection of 0.2–0.4. Blasticidin (10 µg/ml) was applied 24 h after transduction to select stable transductants. Cas9-expressing cells were expanded and transduced with the GeCKOv2 lentiviral library, with puromycin selection (1–1.5 µg/ml) 2–4 days after transduction. For HCT116/54C, 6.4 × 10^7^ cells were transduced with a multiplicity of infection of 0.22 to obtain 1.25 × 10^7^ individual transductants (HCT116/54C GeCKO library; 89% with a single gRNA assuming Poisson statistics), and for UT-SCC-74B, 6 × 10^7^ cells were transduced with a multiplicity of infection of 0.18 to obtain 9.8 × 10^6^ individual transductants (UT-SCC-74B GeCKO library; 91% receiving a single gRNA). Cell populations were expanded for up to 8 weeks and either cryopreserved at scale (2–3 × 10^7^ cells/tube) or used immediately for inoculations.

### Animal experiments

Six- to eight-week-old female NOD scid gamma (NSG; NOD.Cg-*Prkdc*^*scid*^ *Il2rγ*^*tm1Wjl*^*/*SzJ; Jackson Laboratory) or NIH-III mice were bred in the Vernon Jansen Unit, University of Auckland, Auckland, New Zealand. Mice were inoculated with unmodified cell lines at a range of cell numbers, or HCT116/54C or UT-SCC-74B GeCKO libraries (10^7^ cells per site), subcutaneously into bilateral flanks. TD_50_ values, defined as the number of cells required for a 50% probability of tumour development, were determined using logistic regression fitted to the proportion of tumours that developed [36], with a tumour considered to have formed if it reached 250 mm^3^ in volume within 100 days from inoculation. For the pilot study, a small sample size of *n* = 3 per group was chosen. For the larger study, a sample size of *n* = 36 tumours in total was chosen based on available resources. Animals were randomised within blocks to receive treatment with 6-thioguanine (3 mg/kg in PBS qd × 5; Sigma-Aldrich), evofosfamide (50 mg/kg in saline qd × 5/week for 3 weeks; Threshold Pharmaceuticals, San Francisco, CA) or saline (qd × 5/week for 3 weeks) once median tumour volume reached 250 mm^3^. Treatments were administered by intraperitoneal injection at 10 ml/kg. All animals had ad libitum access to food and water in microisolator cages and were maintained on a 12 h light/dark cycle. Animal health and welfare were monitored regularly with animals culled by cervical dislocation or CO_2_ asphyxiation if their condition deteriorated, if they lost in excess of 20% of their pre-manipulation bodyweight or if ethical tumour size limits were reached (longest tumour diameter ≥20 mm). Tumour volume was measured by electronic callipers using the formula *π*/6 × width × length^2^ and was blinded to animal treatment. Differences in tumour growth were assessed by log-rank test on the time taken for tumours to quadruple in size.

### Isolation of genomic DNA

Fifty million cells were harvested from trypsinised cell suspensions, rinsed with PBS and stored at −80 °C. Genomic DNA (gDNA) was isolated using the QIAamp DNA Blood Maxi Kit (QIAGEN, Hilden, Germany). For tumour samples, harvested tumour tissue was cut into small fragments using a scalpel blade and flash-frozen in liquid nitrogen at 0.5 g/tube. Genomic DNA was isolated as described [28] with volumes scaled 1.5× for cells and 3.5× for up to 0.5 g tumour. For tumours >0.5 g, tissue was divided into multiple tubes, and gDNA was isolated from each tube and then pooled. Genomic DNA was desalted by adding NaCl to 0.2 M, followed by cold ethanol to precipitate the DNA and centrifugation at 18,000 × *g* to pellet the DNA. The pellet was rinsed once with cold, freshly prepared 70% ethanol, allowed to air-dry for 10 min and then dissolved in 10 mM Tris, pH 8.0, by heating to 50 °C for 1 h. The gDNA was quality assessed by Nanodrop and agarose gel electrophoresis (0.8%/TBE) and quantified by Qubit DNA-BR assay (ThermoFisher Scientific).

### Polymerase chain reaction

We modified the original PCR protocol designed for lentiGuide-Puro vectors [8, 28], utilising a three-stage PCR protocol to amplify the gRNA sequences from the gRNA cassette in the gDNA in preparation for Illumina sequencing. Herculase II Fusion DNA Polymerase (Agilent Technologies, Santa Clara, CA) was used for all reactions. Each reaction contained 0.5 µl DNA polymerase, 1 mM dNTP mixture, 4% DMSO and 1× Herculase II Buffer in 50 µl volume. Between each PCR, Ampure XP beads (Beckman Coulter, Brea, CA) were used to purify the PCR products, and the Qubit DNA-BR assay was used to quantify products. In PCR1, 0.25 µM of forward and reverse primer (F1 and R1 from reference. [8]) and 5 µg gDNA per 50 µl reaction was used. Cycling parameters were an initial denaturation at 98 °C for 5 min, 20 cycles of denaturation at 98 °C for 60 s, annealing at 62 °C for 60 s, extension at 72 °C for 90 s, and a final extension at 72 °C for 10 min. For each cell culture sample, at least 150 µg was used for PCR1 (split into individual reactions and pooled after PCR1), and for each tumour sample, at least 200 µg (or all gDNA available) was used. PCR2 was used to add the Illumina adapters and barcode the samples, with 10 µl-equivalent of PCR1 mix and 0.2 µM of barcoded primers (PCR2 F Barcode 1–6 and R Barcode 1–6 from ref. [28]) and 6 individual reactions performed for each sample. Cycling parameters were an initial denaturation at 98 °C for 5 min, 10 cycles of denaturation at 98 °C for 45 s, annealing at 62 °C for 35 s, extension at 72 °C for 90 s, and a final extension at 72 °C for 5 min. PCR3 was used to further amplify the product, given low yields and amplification artefacts due to long primers in PCR2. PCR3 was performed with 0.2 µM of primers PCR3F (AATGATACGGCGACCACCGAGATC) and PCR3R (CAAGCAGAAGACGGCATACGAG) and 4 ng of purified PCR2 product. One reaction from pooled PCR2 product was performed. Cycling parameters were an initial denaturation at 98 °C for 2 min, 10 cycles of denaturation at 98 °C for 30 s, annealing at 61 °C for 35 s, extension at 72 °C for 45 s, and a final extension at 72 °C for 10 min. Primers were ordered from Integrated DNA Technologies (Coralville, IA), with HPLC-purified DNA oligos used for PCR1 and PCR3 and desalted Ultramer oligos used for PCR2. Positive controls for PCR used lentiguide-Puro EGFP gRNA (Addgene #80036), which contained a gRNA sequence not present in the GeCKOv2 library. Agarose gel electrophoresis (1.5%/Tris-Borate-EDTA) was used to confirm PCR products (317 for PCR1, ~360 for PCR2/PCR3). Samples were sequenced on a NextSeq500 (Illumina, San Diego, CA) using high-output, 2 × 150 bp flow cells (Illumina).

### Processing of sequencing data

A snakemake-based pipeline for processing sequencing data was developed (https://gitlab.com/twlee79/pooled_screen_counts). Sequencing data were demultiplexed according to the reverse index by the Illumina platform and converted to FASTQ format. These data were then demultiplexed according to the forward index using cutadapt v1.18 with non-internal adapter sequences, allowing up to 1 nucleotide mismatch. Fastqc 0.11.7 was carried out on the demultiplexed reads to check for sequencing quality. Next, cutadapt was used to trim poor quality bases and trim the vector 5′ and 3′ backbone sequence flanking the gRNA sequences. The quality threshold of 10 was used, and the 5′/3′ adapters were specified as linked adapters, allowing an adapter error rate of 0.1 and 19–21 nucleotide resulting sequences (expected 20-nucleotide gRNA sequences); 99.98% of demultiplexed reads were retained after trimming. The gRNA sequences were then aligned to the gRNA sequences present in the GeCKOv2 library using bowtie 2. Replicated gRNA sequences in the GeCKOv2 library were collapsed to individual entries prior to alignment (119,461 unique gRNAs; any replicated gRNA sequences were labelled “non-unique”). Alignment scoring in bowtie 2 was set to local alignment mode with a score of 10 for a match, 4-6 for a mismatch (depending on quality), and per nucleotide gap penalty of −1, reporting the best alignment and also the best alternative alignment score. The number of reads aligned to each gRNA in the library was counted using a custom script (count_fgs_sam; https://gitlab.com/twlee79/count_fgs_sam). The minimum alignment score for a read to be counted was set to 189—allowing for up to two mismatches or a 1-nt gap—with the additional criterion that only unambiguous alignments were counted. An unambiguous alignment was defined as a read where the main alignment had a score of 3 or greater than the best alternative alignment, i.e. the main alignment had fewer mismatches or gaps than the best alternative. A mean of 95.2% of trimmed reads aligned unambiguously and contributed to the counts.

### Read count normalisation

We calculated log normalised counts per million (log-ncpm) using a new normalisation method called MPM—mean of pairwise log expression ratios (M)—that we developed to deal with data containing many zero counts on the low end and inflated counts on the high end (Supplemental Methods). As the GeCKOv2 gRNA library contains 1000 non-targeting controls (NTCs), which can be assumed to be neutral, and thus non-differential on average, we calculated the size factors from NTC gRNAs alone with no *a* or *m* trimming (*a* = 0, *m* = 0), and a pseudocount of 0.02. The normalised counts thus represent counts relative to neutral gRNAs. Unless stated otherwise, pseudocounts are used for normalisation only, and we present log_2_ normalised counts per million as-is without a pseudocount (zeroes at negative infinity; plotted at *y*-axis minimum).

### Analysis of gRNA counts with edgeR

The edgeR function *estimateDisp* was used to estimate the dispersion of gRNA counts with MPM-normalised library sizes. The gRNA data was filtered to retain only gRNAs detected in at least 6 large tumour samples and with at least 12 reads in total from the large tumour samples. The dispersion was estimated in edgeR ‘classic’ mode (using the quantile-adjusted conditional maximum likelihood method) for a single-factor experiment (with a level of the factor for each experimental condition) [37]. Hypothesis testing between experimental groups at the gRNA level was performed using the edgeR *exactTest* function. Gene-level analysis was conducted using the α-RRA method [38] with gRNAs ranked by edgeR *p*-value and α set to a false discovery rate (FDR) cutoff of 0.2. RRA was conducted separately for positive (i.e. positive log fold change (FC) vs. control) and negative selection (negative logFC vs. control).

### Estimation of the number of clones in tumour samples

All cells were assumed to have an independent and equal probability *p* of surviving to form a clone. If *n*_*i*_ is the number of cells containing gRNA *i* that are inoculated, the detection of that gRNA was modelled as the outcome of a set *n*_*i*_ Bernoulli trials. Under these assumptions, the probability of the gRNA being not detected is given by ${\left(1-p\right)}^{{n}_{i}}$ (i.e. the probability that no cells with the gRNA survive), and the probability of the gRNA being detected is given by $1-{\left(1-p\right)}^{{n}_{i}}$ (i.e. the probability that at least one cell with the gRNA survives to form a clone). A likelihood function was defined based on the joint probability for the observed detection of a given subset of gRNAs (non-targeting or all gRNAs), and *p* and its variance was estimated using maximum likelihood estimation, with *n*_*i*_ parameterised by the proportion of counts for gRNA *i* in the inoculum multiplied by the number of cells inoculated. The median number of surviving clones per gRNA was then estimated as the median of $p{n}_{i}$ across all gRNAs. To account for the variable number of counts in each sample, the same detection threshold (1 count) was used across all samples, but we subsampled to a target NTC count of 10^5^ counts (approximately 7 million total counts) prior to estimating *p*. To reduce the dependence of the estimate on particular subsamples, a total of 99 subsamples per sample was performed, and the median was used. Three large tumour samples had fewer than 10^5^ NTC counts and were excluded.

### Simulations of reduced library sizes

To generate datasets containing fewer gRNAs, we randomly combined gRNAs into bins of 2, 5, 10, 20, 50, 100, 200, 400, 800 and 1600 gRNAs, with binning performed separately for targeting and non-targeting gRNAs; the counts of any excess gRNAs that could not fill a full bin were discarded. The binned gRNAs were further subsampled to 100 000 (no binning), 50 000 (bin size = 2), 20 000, 10 000, 4000, 2000, 1000, 500, 250, 125 and 62 (bin size = 1600) gRNAs to reduce the dependence on particular gRNAs. A total of 100 simulated datasets were generated for each number of gRNAs. We conducted analyses on either combined small tumours (total *n* = 11 combining pilot HCT116/54C tumours and 14 d tumours in treatment experiment) or large tumours (total *n* = 28 combining no drug, 6-thioguanine and evofosfamide groups), with a varying number of tumour samples randomly drawn from each dataset to assess accuracy and precision of estimating dispersion from datasets of different sizes. Data sets were normalised by MPM (*a* = 0, *m* = 0, pseudocount = 0.02) based on subsampled NTC gRNAs (bin sizes ≤ 50) or all subsampled gRNAs (bin sizes > 50). The common dispersion was calculated using edgeR in ‘classic’ mode with all samples in a single group. Power analyses were conducted on the RnaSeqSampleSize package [39] using the *estPower* function with dispersion (phi) set to the common dispersion estimate. For all comparisons, mean count (lambda) was set to 10, and default options of 1 for normalisation factor and sample size ratio, FDR of 0.05, and 1% prognostic genes. Power was calculated for various combinations of sample size and effect size.

## Results

### Selection of suitable cell lines for in vivo CRISPR/Cas9 screens

**Fig. 1:**
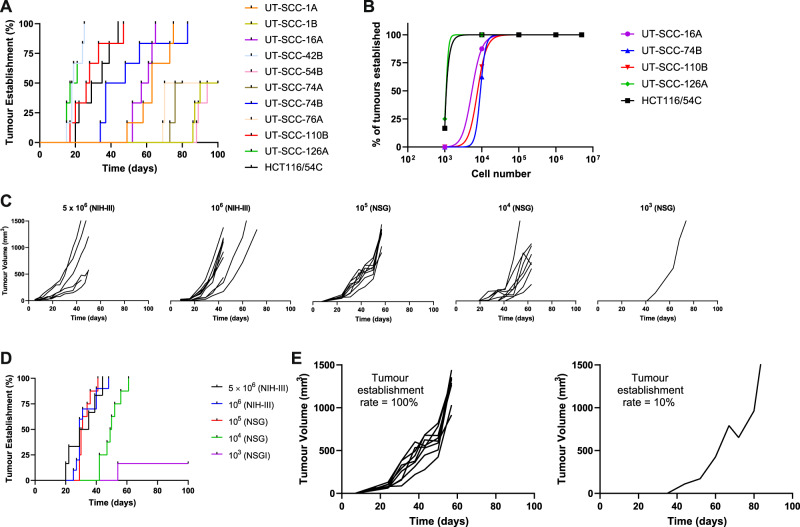
Establishment of tumour xenograft models. **A** Rate of tumour establishment for UT-SCC and HCT116/54C cell lines inoculated bilaterally in NIH-III mice at 5 × 10^6^ cells per flank. **B** Frequency of tumour establishment for UT-SCC and HCT116/54C cell lines inoculated at different cell inocula into NIH-III (≥10^6^) or NSG (≤10^5^) mice. **C** Comparison of HCT116/54C tumour growth in NIH-III and NSG mice at different cell inocula. **D** The rate of tumour establishment for HCT116/54C at different cell inocula. **E** Comparison of tumour establishment rate and growth for HCT116/54C tumours in NSG (left panel) and NIH-III (right panel) mice inoculated with 10^5^ cells.

We hypothesised that a cell line with a high proportion of tumour-initiating cells would be better suited for in vivo CRISPR/Cas9 screens as it would undergo a reduced population bottleneck due to a larger founder population and therefore be less sensitive to stochastic effects. We evaluated 22 UT-SCC head and neck squamous cell carcinoma cell lines and an HCT116 colon carcinoma subline (HCT116/54C) by inoculating immunodeficient mice subcutaneously with different numbers of cells to identify which cell lines could initiate tumours with few cells. In initial experiments, only 11 of 23 cell lines grew as tumour xenografts within 100 days of inoculation of 5 × 10^6^ cells into NIH-III mice (Fig. 1A). Four of these cell lines (UT-SCC-1B, UT-SCC-54B, UT-SCC-74A, and UT-SCC-76A) had low tumour take rates and slow growth with ≤50% of tumours established by day 90, while two others (UT-SCC-1A and UT-SCC-42B) had frequent ulceration of skin over the tumours.

The five cell lines with suitable growth kinetics (UT-SCC-16A, -74B, -110B, -126A and HCT116/54C) were selected to conduct limiting dilution assays in NIH-III and NSG mice to estimate the TD_50_. We initially screened all cell lines for growth as tumour xenograft models in NIH-III mice by inoculating 5 × 10^6^ cells per flank into 3–5 mice, followed by 10^6^ and 10^5^ cells for those with a high take rate (>75%). For tumour models that grew as xenografts at 10^5^ or 10^6^ cells, we proceeded to test in NSG mice, which were expected to be more conducive to tumour growth, at 10^5^, 10^4^ and 10^3^ cells until fewer than 50% of tumours formed. Tumours were formed in 100% of NIH-III mice at ≥10^6^ for all cell lines, but the establishment rate fell with cell inocula ≤ 10^4^ in NSG mice (Fig. 1B). As expected, tumour growth took longer on average with smaller inocula (Fig. 1C, D) and tumour establishment rate was higher and faster in NSG mice than NIH-III mice (Fig. 1E). The lowest TD_50_ of approximately 1100 cells was estimated for HCT116/54C and UT-SCC-126A, followed by UT-SCC-16A, UT-SCC-110B and UT-SCC-74B (Table 1). Despite its low TD_50_, UT-SCC-126A was not investigated further because it caused considerable weight loss in some animals (e.g. three of four NSG mice inoculated bilaterally with 10^4^ UT-SCC-126A required culling early due to ~20% bodyweight loss).

**Table 1:** Cell line characteristics and TD_50_.

Cell line	Primary site	Type	TD50*
HCT116/54C	Colon	Primary	1100
UT-SCC-126A	Labii inferioris	Primary	1100; mice lose weight
UT-SCC-16A	Lingus	Primary	5400
UT-SCC-110B	Gingiva, maxillary sinus	Metastasis	7700
UT-SCC-74B	Lingus	Recurrent	9200
UT-SCC-1A	Gingiva	Primary	<10^6^; ulcerates
UT-SCC-42B	Supraglottic larynx	Metastasis	<10^6^; ulcerates
UT-SCC-1B	Gingiva	Recurrent	Low take rate
UT-SCC-54B	Buccal mucosa	Recurrent	Low take rate
UT-SCC-74A	Lingus	Primary	Low take rate
UT-SCC-76A	Lingus	Primary	Low take rate
UT-SCC-16B	Lingus	Metastasis	Did not grow
UT-SCC-19A	Glottic larynx	Primary	Did not grow
UT-SCC-19B	Glottic larynx	Recurrent	Did not grow
UT-SCC-24A	Lingus	Primary	Did not grow
UT-SCC-42A	Supraglottic larynx	Primary	Did not grow
UT-SCC-46A	Gingiva, maxilla	Primary	Did not grow
UT-SCC-54A	Buccal mucosa	Primary	Did not grow
UT-SCC-59C	Parotid	Metastasis	Did not grow
UT-SCC-60A	Tonsilla	Primary	Did not grow
UT-SCC-63A	Gingiva, mandibula	Primary	Did not grow
UT-SCC-76B	Lingus	Recurrent	Did not grow
UT-SCC-110A	Gingiva, maxillary sinus	Recurrent	Did not grow

^a^Low take rate indicates ≤50% of tumours established by day 90 at 5 × 10^6^ cells in NIH-III mice. Did not grow indicates no tumours were established by day 90 at 5 × 10^6^ cells in NIH-III mice.

### Pilot xenograft experiments with HCT116/54C and UT-SCC-74B GeCKOv2 libraries

**Fig. 2:**
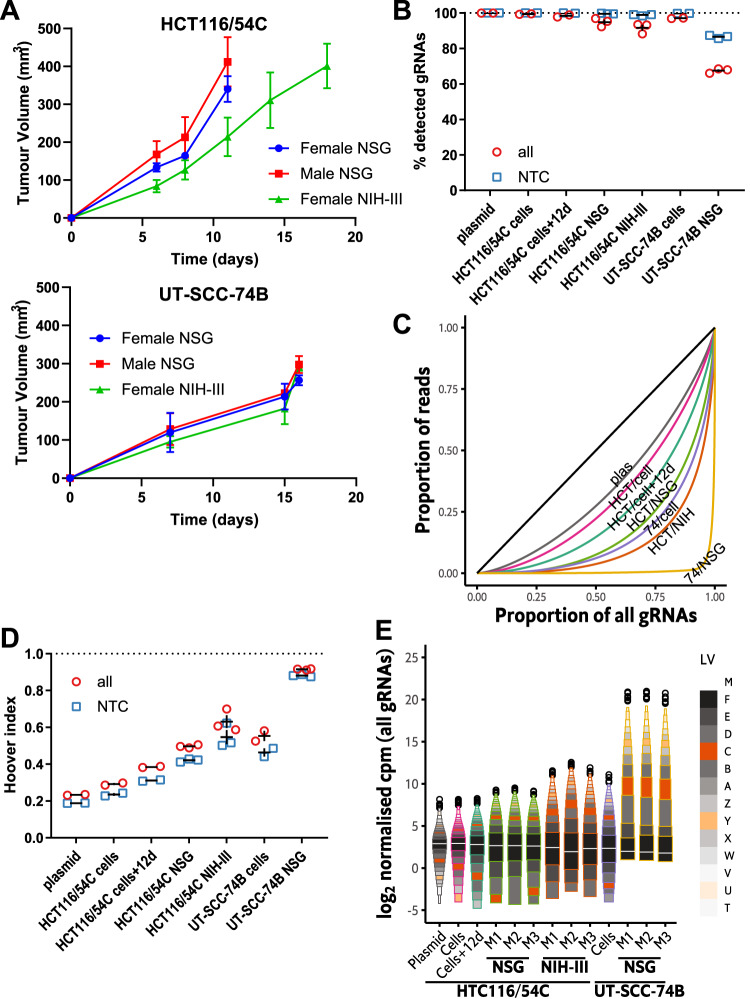
Pilot whole genome in vivo screens for HCT116/54C and UT-SCC-74B GeCKOv2 libraries. **A** Growth curves for HCT116/54C and UT-SCC-74B tumours in NSG and NIH-III mice. Bars represent the mean and SEM of 3–6 tumours. **B** Percentage of all or NTC gRNAs detected for plasmid, HCT116/54C GeCKO cell inoculum, HCT116/54C GeCKO tumours in NSG mice, HCT116/54C GeCKO tumours in NIH-III mice and UT-SCC-74B GeCKO cell inoculum and tumours in NSG mice. Replicates for plasmid and cell samples are PCR replicates from the same gDNA sample (*n* = 2 each); tumour replicates are individual tumours in separate hosts (*n* = 3 per group). The bar indicates mean ± SEM. **C** Lorenz curves to show the distribution of all gRNA read counts. The curve for summed PCR replicates (plasmid and cell samples) or median tumours (by Hoover index) in a group is highlighted. The black line is the line of equality (plas: plasmid; HCT: HCT116/54C; 74: UT-SCC-74B; NIH: NIH-III). **D** Hoover index of each sample as a measure of inequality of the read counts for all and NTC gRNAs. **E** Letter-value plot of log normalised counts per million (log-ncpm) for all gRNAs in plasmid, cell inocula and GeCKO tumour samples (zeroes given pseudocount of 0.5). The white line inside the black boxes indicates the median (*M*), and the black boxes indicate the upper/lower quartiles (*F*; fourths). The next smallest boxes indicate the upper/lower eighths (*E*) and so on. Counts for plasmid and cell samples were summed from two PCR replicates prior to normalisation. The remaining outliers are shown as open circles.

We next compared tumour xenografts of GeCKOv2 libraries of HCT116/54C (joint lowest TD_50_) and UT-SCC-74B (a second model with a higher TD_50_) cell lines to ascertain whether the TD_50_ was consistent with model suitability for in vivo screens. Two mouse strains—NSG and NIH-III mice—were compared by inoculating 10^7^ cells and harvesting tumours when the smallest in each group reached ~250 mm^3^. HCT116/54C GeCKO tumours grew faster in NSG mice than NIH-III mice and faster than UT-SCC-74B GeCKO tumours, which grew equally in both mouse strains (Fig. 2A).

Almost all gRNAs were detected in the GeCKOv2 plasmid library (99.98%), the HCT116/54C GeCKO library used for inoculation (99.7%), and cells cultured in vitro for a further 12 d (99.3%; Fig. 2B; Table S1). We observed high representation in HCT116/54C GeCKO tumours in NSG hosts with 92.2–96.9% gRNAs detected, with slightly lower representation in NIH-III hosts at 88.3–93.5% (Fig. 2B). The vast majority of gRNAs were also detected in UT-SCC-74B GeCKO cells (98.5%) but UT-SCC-74B GeCKO tumours in NSG mice had considerably lower representation of 66.0–68.5%, with many detected with only a single read (Fig. 2B; Table S1). In considering only NTC gRNAs, >98% were detected in all HCT116/54C GeCKO tumours but only ~86% in UT-SCC-74B GeCKO tumours (Fig. 2B; Table S2). This suggests that cells receiving non-targeting gRNAs had a growth or survival advantage within the tumours compared to cells receiving most gRNAs targeting a gene.

The decreases in representation in the tumour samples were accompanied by disproportionate increases in the read counts of the top-ranked gRNAs (i.e. those with the highest counts; Table S1). The unequal read count distributions in the tumour samples were also evident in the Lorenz curves of the gRNA count data (Fig. 2C), with the UT-SCC-74B GeCKO/NSG tumours showing extremely unequal read counts (top 1% of gRNAs accounting for 76–78% of reads). The NTC gRNAs showed only slightly smaller read count inequality than gene-targeting gRNAs, suggesting that the major driver is stochastic effects rather than changes in fitness (Table S2, Fig. S1A). As a metric of inequality, we calculated the Hoover index, which can be interpreted as the proportion of reads needed to be redistributed to form a uniform distribution (Fig. 2D; Tables S1 and S2). The Hoover indices followed the inverse pattern of gRNA representation (compare Fig. 2D to B). The inverse relationship suggests that the apparent loss of gRNA representation is at least partly driven by difficulty sampling low-frequency gRNAs in highly unequal gRNA distributions.

The skewed distribution of read counts, especially in UT-SCC-74B tumours, meant that the data were zero-inflated at one end and contained very high counts for a few gRNAs at the other end. We found that most count normalisation methods, including the commonly used TMM [40] and RLE [41] methods, as well as the GMPR method [42] developed for zero-inflated microbiome data, performed poorly with these highly unequal count distributions containing many sampling zeroes. Therefore, we devised a new normalisation procedure, which we call MPM, for our data to account for its unique characteristics (Supplemental Methods and Results; Figs. S4–S7). MPM is a generalisation of the GMPR method, but a key difference is the treatment of zeroes—these are excluded completely in GMPR but are substituted in MPM for pseudocounts when a zero is present in one sample during a pairwise comparison (double zeroes are excluded). This treatment is more consistent with all zero counts being modelled as sampling zeroes, as suggested in previous work [43]. The exclusion of all zeroes in GMPR results in the loss of information that a count is lower in one sample than another sample and can bias library size estimates upwards; MPM using an appropriate pseudocount value performs better in this regard (see Supplemental Results).

Following MPM normalisation, each sample had similar median normalised gRNA counts (Fig. 2E; Fig. S1B; Fig. S9; Tables S1 and S2). This data representation again reveals that the loss of representation from the plasmid to the cell libraries and the tumours is accompanied by wider, more skewed gRNA distribution, with the upper tails becoming progressively more extreme.

### Increased read count inequality following subsequent growth of HCT116/54C GeCKO tumours

**Fig. 3:**
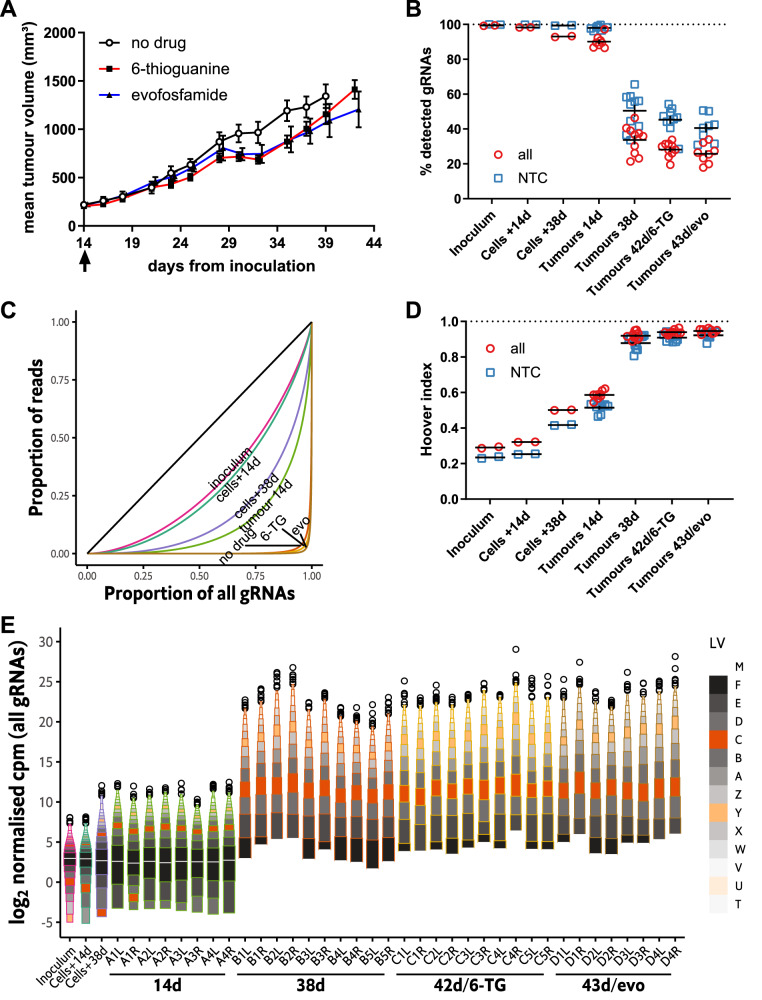
Whole genome in vivo screens for HCT116/54C GeCKOv2 libraries in small and large tumours. **A** HCT116/54C GeCKO tumour growth after the commencement of drug treatment at day 14 (arrow) (mean ± SEM; *n* = 8–10). **B** Percentage of all or NTC gRNAs detected for HCT116/54C GeCKO cells (inoculum or cultured an additional 14 or 38 days after inoculation) and HCT116/54C GeCKO tumours collected at 14 days, 38 days with no drug, 42 days with 6-thioguanine (6-TG) or 43 days with evofosfamide (evo). Replicate cell samples are PCR replicates from the same gDNA sample (*n* = 2 each); tumour replicates are individual tumours grown bilaterally (*n* = 8 for 14 d and evo; *n* = 10 for 38 d and 6-TG). The bar indicates mean ± SEM. **C** Lorenz curves to show the distribution of all gRNA read counts. The curve for summed PCR replicates (plasmid and cell samples) or median tumours (by Hoover index) in a group is highlighted. The black line is the line of equality. **D** Hoover index of each sample as a measure of inequality of the read counts for all and NTC gRNAs. **E** Letter-value plot of log normalised counts per million (log-ncpm) for all gRNAs in cell and tumour samples (zeroes given pseudocount of 0.5). Counts for plasmid and cell samples were summed from two PCR replicates prior to normalisation. Sample name refers to group, animal number and flank position (e.g. A1L represents a tumour on the left flank of mouse 1 from group A).

Using the best combination of cell line and mouse host, we evaluated the feasibility of conducting an in vivo screen with this model. HCT116/54C GeCKO tumours (*n* = 36) in female NSG mice were allowed to grow until the median tumour size reached ~250 mm^3^ then the mice were randomised into four groups: one (A) in which tumours were harvested immediately, a mock treatment group (B) and two drug treatment groups (C with 6-thioguanine, 6-TG and D with evofosfamide). Tumours in B-D were allowed to grow until the largest tumour in each group reached ethical size limits, at which point all tumours in the group were collected (day 38, 42 and 43 post inoculation for groups B, C and D, respectively). There was a small non-significant decrease in tumour growth for the two drug treatment groups compared to controls (Fig. 3A). Representation of gRNAs in the group A tumours was high (90.1 ± 3.5%; mean ± SD) but was lower than in the similarly-sized tumours in the pilot study (94.8 ± 2.8%), most likely reflecting that the tumours grew slightly slower than in the pilot study, taking 14 days to reach an average size of 230 mm^3^ compared to 11 days to reach an average of 340 mm^3^. Moreover, there was a substantial decrease in representation of all gRNA and NTC gRNAs in tumours in groups B–D relative to the group A tumours at day 14 (Fig. 3B; Table S3). A much smaller loss of representation was observed relative to the cell inoculum by keeping the cells used for inoculation in culture for the duration of the in vivo screen (95.0% gRNAs detected after 38 additional days).

Similar to the small tumours in the pilot study, the decreases in gRNA representation were mirrored by increases in inequality and skew in the gRNA counts. Lorenz curves and Hoover indices show inequality was much greater in the 14-day tumours (Hoover indexes 0.54–0.62) compared to the cell inoculum (0.29) or cells cultured for an additional 14 days after inoculation (0.32) and somewhat greater than that of cells cultured for an additional 38 days after inoculation (0.50; Fig. 3C, D; Table S3). All large (38–43 day) tumours had extremely unequal gRNA distributions as shown by the Lorenz curves, Hoover indices of 0.89–0.96, and 74–96% of reads from only the top 1% of gRNAs. Letter-value plots of normalised gRNA counts similarly showed distribution becoming progressively wider going from the cell culture samples to the 14-day tumours and then large (38–43 day) tumours (Fig. 3E; Fig. S10). The latter gRNA distributions all had very long upper tails indicating a strong skew towards relatively few gRNAs having very high counts, while most others had zero or very few counts. In the most skewed large tumour sample (C4R), 17.6% of reads were from a single gRNA (Table S3). The median gRNA count was zero for all large tumours. As with the pilot study, similar but slightly smaller increases in count inequality and skew could be seen in an analysis of NTC gRNAs in the tumour samples (Fig. 3B, D; Fig. S1C, D; Table S4).

### Random variation in clonal dynamics precludes gRNA-induced selection in HCT116/54C GeCKO tumours

**Fig. 4:**
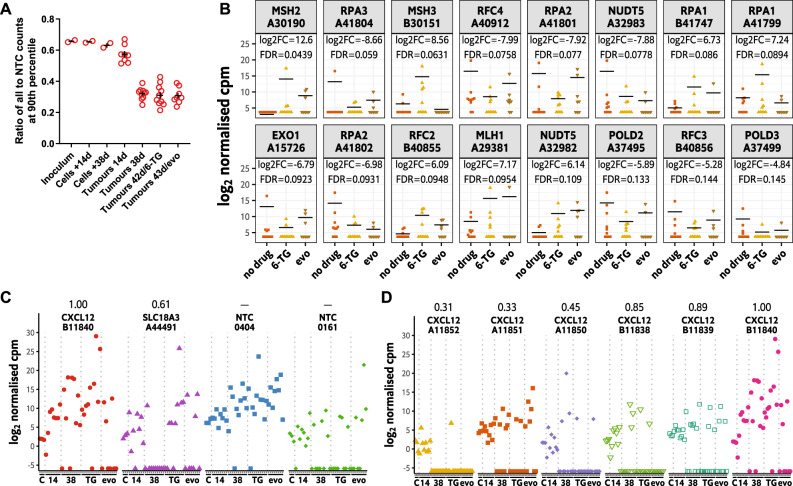
Assessing gRNA-driven selection in HCT116/54C in vivo screens. **A** Ratio of counts for the gRNA at the 90th count percentile among all gRNAs to counts for the NTC gRNA at the 90th count percentile. The bar indicates mean ± SEM. **B** Distribution of log_2_ normalised cpm of the top 16 gRNAs (by *p*-value control versus 6-thioguanine) targeting genes expected to be involved in 6-thioguanine resistance in the tumour samples. The selected genes were *HPRT1*, *NUDT5* and 23 genes in the KEGG mismatch repair geneset (6-TG = 6-thioguanine, evo = evofosfamide). Lines are the average log cpm calculated with *aveLogCPM* (prior count of 0.5), and statistics are for the no drug versus 6-thioguanine comparison using the edgeR *exactTest* function with dispersion estimated from large tumour samples only. **C** Variation in counts of four gRNAs across samples in the large tumour dataset. The four gRNAs represent those with the highest and 10th highest maximum log normalised cpm across all samples for all or NTC gRNAs. **D** Variation in counts of the six gRNAs in the library targeting *CXCL2* across samples in the large tumour dataset. For **C**, **D**, the numbers above each individual targeting gRNA represent previously reported estimates of guide activity [14], with scores approaching 1 representing highly effective gRNAs and those approaching 0 representing poorly performing gRNAs.

The representation and count distribution data in HCT116/54C GeCKO tumours suggested a greater loss of targeting gRNAs compared to non-targeting controls. To more directly test this, we compared the ratio of the 90th percentile read counts of all gRNAs to the 90th percentile read counts of the NTC gRNAs. The 90th percentile was used to obtain a reasonable number of counts since the median and upper quartile were zero for several large tumour samples. If the distribution of read counts for all gRNAs and NTC gRNAs was changing uniformly during tumour growth, this ratio was expected to stay constant for all groups. Instead, there was a progressive decrease in this ratio from the cell samples to the small tumours (14 d) to the large (38–43 d) tumours (Fig. 4A). These data indicate a greater depletion of cells harbouring targeting gRNAs compared to NTC gRNAs in the tumours. A likely explanation of this effect is the overall negative selection of cells harbouring knockouts compared to control cell lines due to a general loss of fitness in these cells. Thus, although there were large random differences in growth and survival among clones, some effects of selection could be detected when averaging across a large number of gRNAs.

Given the extremely high variability in the large tumour counts, we reasoned that detection of any gRNA-driven selection among the treatment groups would be rare. Unexpectedly, after filtering for low abundance gRNAs (73,421 targeting gRNAs retained), an initial analysis of the entire in vivo screen data set (including small 14 d tumours) using the edgeR exact test pipeline [37] resulted in the detection of a large number of gRNAs significantly selected either positively or negatively (>10,000 each direction; FDR < 0.1) for the comparisons between untreated vs. 6-TG and untreated vs. evofosfamide. Further investigation revealed that this was caused by the inclusion of the small tumours during the analysis. Including only the large tumours in the analysis resulted in the detection of a more realistic number of gRNAs as being selected (approximately 2000 in each direction for 6-thioguanine and 0 for evofosfamide; FDR < 0.1; Supplemental Data Files 1 and 3). This was due to a change in the dispersion estimates—common dispersion was 4.8 for the dataset including small tumours, and 8.2 for the large tumours only. In the first analysis, the inclusion of small tumours, which had lower variability in counts, effectively biased the estimated dispersion downwards relative to what would be applicable for the large tumours, resulting in failure to control type I error.

Although a number of gRNAs were detected at FDR < 0.1 in the large tumour dataset for 6-thioguanine, gene-level analysis of these gRNA-level data by RRA [38] detected no gene hits at FDR < 0.1 for either 6-TG or evofosfamide compared to untreated (Supplemental Data Files 2 and 4). To further evaluate the performance of the screen, we assessed the enrichment of gRNAs targeting well-characterised 6-TG sensitivity genes *HPRT1* and *NUDT5* [44], as well as those in the mismatch repair pathway [7], in treated tumours. The top gRNAs (by FDR) targeting this subset of genes were either enriched or depleted in treated tumours vs. controls rather than being predominately enriched (Fig. 4B). For example, one gRNA targeting *NUDT5* was depleted (A32983 log_2_ FC − 7.9, FDR = 0.078), but another was enriched (A32982 log_2_ FC 6.1, FDR = 0.11). In addition, there was large variability in gRNA counts and frequent incidence of zero counts across all tumours, such that any apparent enrichment in one group versus another was largely driven by a small number of tumours having very high counts despite other samples having much lower counts (potentially zero).

The high count dispersion, together with the pattern of read counts among individual tumours for a particular gRNA, suggest a strong random component of clonal expansion within each tumour. Individual clones (with gRNAs as barcodes) potentially expand to a very high frequency in an apparently random fashion, which masks any potential effects of selection. Indeed, we found that the identities of the top gRNAs were different in each sample (Fig. S2A), and Spearman correlation analysis failed to identify any grouping of large tumours by treatment group or overall (Fig. S2B). To further demonstrate this, we plotted the read counts of the gRNAs with the highest (*CXCL12* gRNA #B11840) and 10th highest (*SLC18A3* gRNA #A44491) log-ncpm across all samples, as well as the NTC gRNAs with the highest (NTC gRNA #0404) and 10th highest (NTC #0161) log-ncpm across all samples. The read counts of these four gRNAs varied widely across large tumour samples (Fig. 4C). For instance, the log-ncpm for *CXCL12* gRNA #B11840 in one 6-TG-treated tumour was 26.5, while zero counts were detected in another 6-TG-treated tumour. This wide variation suggests largely random expansion of clones within these tumours. In smaller tumours, the counts were less variable but were still unusually high or low in certain samples, indicating a degree of random clonal expansion was already present in these smaller tumours. To confirm this variation applied across all gRNA targeting the same gene, we also investigated all gRNAs targeting *CXCL12* and observed a similar level of variation between samples as we had seen for *CXCL12* gRNA #B11840 (Fig. 4D). Estimated guide activities (in the range 0–1; ref. [14]) for the *CXCL12* gRNAs vary from 0.31 to 1.00. Although the *CXCL12* gRNA with the most variable counts (#B11840) was the one with the highest estimated activity (1.00) while the least active guide (#A11852; 0.31) had the most dropouts, the other guides with estimated activities in the range 0.33–0.89 showed a similar pattern of count variability. This is consistent with the variation in counts being driven by stochastic effects instead of gRNA-editing effects.

### Count dispersion increases with growth time and is much greater in tumour samples

**Fig. 5:**
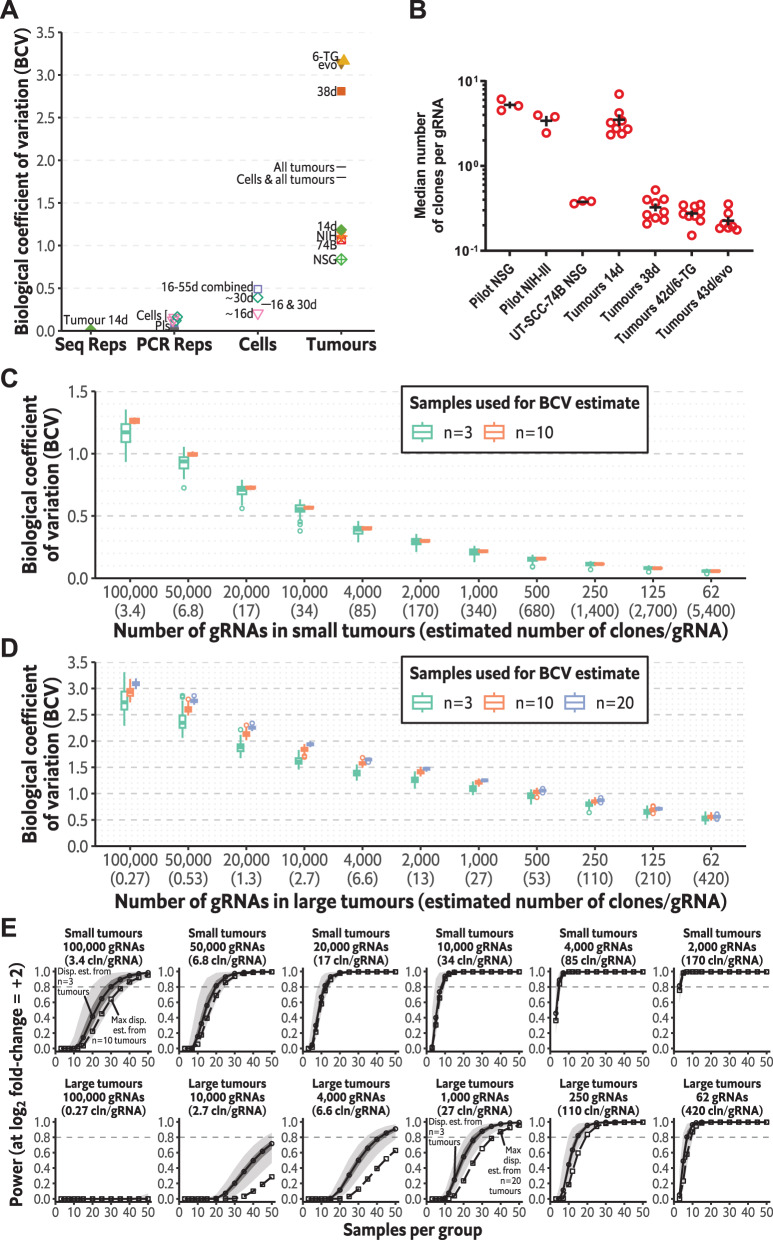
Count dispersion and statistical power in HCT116/54C whole genome and simulated libraries of reduced size. **A** Biological coefficient of variation (BCV) estimated in edgeR for different subsets of samples. Data points represent different sample groups, and lines represent combinations of sample groups. Seq Reps: Sequencing replicates (*n* = 2) of the same library (14 d tumour) produced by PCR. PCR Reps: PCR replicates of the plasmid DNA (Pls) or the same genomic DNA samples from cultured HCT116/54C GeCKO cells (Cells), *n* = 2 each. Cells: Replicate HCT116/54C GeCKO cell samples cultured in parallel from the time zero sample for 16–17 d (~16 d; *n* = 2), 28–31 d (~30 d; *n* = 2), or all samples combined (*n* = 5), including one sample cultured for 55 d; any PCR replicates were collapsed by summing prior to calculating BCV for these biological replicates. Tumours: Pilot study tumours for HCT116/54C GeCKO in NSG (NSG, *n* = 3) and NIH-III mice (NIH; *n* = 3) and UT-SCC-74B GeCKO in NSG mice (74B, *n* = 3). Larger study tumours collected early (14 d, *n* = 8), control-treated tumours (38 d, *n* = 10), 6-thioguanine-treated tumours (6-TG; *n* = 10) and evofosfamide-treated tumours (evo; *n* = 8). **B** Estimates of the median number of clones present in each sample from the pilot and larger study, based on the estimated probability of survival of cells with neutral (NTC) gRNAs, and the median number of cells per gRNA. **C** Estimates of BCV in simulated datasets of reduced library size by random binning and/or subsampling gRNAs and samples from small tumour data (NSG pilot + 14 d). Boxplots show the distribution of the BCV estimates for 100 simulated datasets for *n* = 3, *n* = 10 or *n* = 20 tumours and indicate the number of gRNAs. **D** Estimates of BCV in simulated datasets of reduced library size from large tumour data (untreated, 6-TG and evo; 38–43 d). The number of clones/gRNA in **C** and **D** is estimated based on the median number of clones/gRNA for a full library multiplied by the binning factor. **E** Power curves determined from the common dispersion estimates of simulated datasets at a log_2_ effect size of 2 for small and large tumours. Circles and solid lines indicate the curve obtained from the dispersion estimate from *n* = 3 tumours; the median across 100 simulated datasets is plotted. The dark grey area represents the lower and upper quartiles, and the light grey area is the range. The squares and dashed line indicate the power curve when using the maximum dispersion estimate across 100 simulated datasets from *n* = 10 (small tumours) or *n* = 20 (large tumours); cln/gRNA: clones/gRNA.

To obtain a statistical measure of the level of random variation in gRNA counts in different sample groups, we used edgeR to estimate the negative binomial dispersion for different subsets of our dataset. To simplify comparisons, we determined the common dispersion, which assumes all gRNAs have the same dispersion, and took the square root of this to estimate the biological coefficient of variation (BCV; square root of common dispersion) for each subset. We performed this analysis on both technical replicates and biological replicates. Among technical replicates, the BCV across sequencing runs for the same sample was low at 0.012 (Fig. 5A), which is expected given this variation should follow a Poisson distribution. BCV of a similar magnitude was obtained for PCR replicates of the GeCKOv2 plasmid sample (0.022), but interestingly the BCV for PCR replicates of various samples of HCT116/54C GeCKO cells was greater (0.091–0.164), suggesting amplification bias during the PCR process used for gRNA readout was contributing to count variation. Several HCT116/54C GeCKO cell samples collected at different times were available, with culture periods from the initial post-transduction sample (‘time zero’) varying from 16–55 d. When all cell samples (*n* = 5) were analysed as a single group, the BCV was 0.489, while cell samples cultured ~30 d (28–31 d, *n* = 2) had a BCV of 0.392 and those cultured ~16 d (16–17 d, *n* = 2), 0.208, showing that dispersion was increasing with growth time. The BCV of even small tumour samples was considerably greater at 0.84–1.18, while for the large tumours, it increased further to 2.81-3.17. These increases in dispersion with growth in both the cell and tumour samples are consistent with the widening of the count distribution that occurred with increased growth time (Fig. 3E). We also estimated the count dispersion for various combinations of sample groups to illustrate the risk of underestimating dispersion when a lower dispersion group is included among higher dispersion groups. The BCV with all large tumour groups was 2.83, but when 14 d tumours were included, this decreased to 2.16, further decreasing to 1.92 if the pilot tumours were also included. Combining cell samples and all tumours led to additional decreases in the BCV estimate (Fig. 5A).

### Simulations show reduced count dispersion in tumour samples with reduced gRNA library sizes

The GeCKOv2 whole genome gRNA library used in this study consists of 119 461 unique gRNAs. With 10^7^ cells inoculated per tumour, there was an average of 84 cells/gRNA. However, not all these cells will survive to form a clone. To approximate the number of clones/gRNA, we used a binomial model to estimate the overall probability of a cell with neutral (NTC) gRNA surviving in a tumour to form a clone based on the pattern of gRNAs detected (at a given detection level) and the count distribution of the cell inocula. For small NSG tumours, the estimated probability of a cell surviving to form a clone was 0.034–0.10 (1 in 10–30), which implies a median of 2.3–7.0 clones/gRNA (Fig. 5B). In large tumours, the probability of clones continuing to survive decreases to 0.0022–0.0074 (1 in 134–459), implying a number of surviving clones/gRNA of 0.15–0.52 (Fig. 5B). If we assume that the number of clones/gRNA is a more important metric for coverage in in vivo screens compared to cells/gRNA, these estimates suggest that the effective in vivo coverage of our library was several orders of magnitude less than typically recommended for in vitro screens (minimum coverage of at least 100 cells/gene is recommended; ref. [45]).

These estimated numbers of clones/gRNA suggest very low effective in vivo coverage of our library. Improving coverage by increasing the number of cells inoculated per gRNA would result in greater averaging of the random effects on growth and survival, resulting in reduced count dispersion, thereby increasing statistical power to detect gRNA selection. Although there was limited scope to increase the total number of cells inoculated, the number of cells inoculated per gRNA could be increased by reducing the total library size. To assess the effects of using a smaller gRNA library for in vivo screens, we simulated smaller gRNA libraries by randomly combining the gRNA counts in our full-size library into bins of 2–1600 gRNAs, followed by subsampling gRNAs (and samples) to 100,000 (unbinned), 50,000, 25,000, 10,000, 4000, 2000, 1000, 500, 250, 125 or 62 gRNAs; these represented datasets with coverage 84–130,000 cells/gRNA, respectively, but with much lower coverage in terms of clones/gRNA: 3.4–5400 clones/gRNA in small tumours, 0.27–420 clones/gRNA in large tumours, based on our estimates of the number of clones/gRNA in our full library. We reasoned that our binned counts would mimic the random characteristics of clonal expansion in smaller libraries, as clones with different gRNAs in the larger library would be largely equivalent (in terms of random characteristics) to different clones that receive the same gRNA in a smaller library. Any signal due to the gRNAs themselves, which we have already established was small relative to the random clonal expansion effect (Fig. 4), was further averaged out by the random nature of the binning, particularly among bins with a greater number of gRNAs, as well as subsampling to reduce dependence on any particular sample or gRNA.

To assess the effects of a smaller gRNA library on count dispersion and statistical power, we estimated the common dispersion using edgeR following binning/subsampling on our dataset. The dispersion was estimated with a varying number of tumour samples to evaluate the degree to which the dispersion that would apply to a large dataset could be estimated from only a small number of samples; such a situation would occur, for example, when a pilot experiment is conducted to inform power calculations for a later full-sized experiment. For small tumours, our simulations showed that a reduction in BCV to approximately 0.5 could be achieved with 10,000 gRNAs, a further reduction to about 0.3 with 2000 gRNAs, which are of a similar BCV magnitude to samples of HCT116/54C GeCKO cells, and below 0.1 with very small library sizes of <200 gRNAs (Fig. 5C). When these library sizes were considered in terms of the estimated number clones/gRNA, BCV values of 0.15–0.3 were achievable with ~150–700 clones/gRNA in small tumours. For large tumours, a reduction in gRNA library size to 2000 or less reduced the BCV below 1.5, similar to in small tumours with a full library, while a very small library size of 62 gRNA was required to achieve a similar BCV to in cells of approximately 0.5 (Fig. 5D). On a per clone basis, dispersion as estimated by BCV was also higher in large tumour samples compared to small tumour samples, which is consistent with random clonal dynamics being cumulative throughout the tumour growth period. BCV estimates were less precise when determined from a small number of pilot samples, as indicated by the greater range, particularly at the lower end, when estimated from *n* = 3 compared to using a larger number of samples (*n* = 10 or *n* = 20), coupled with a tendency for the median to be lower at *n* = 3 (Fig. 5C, D).

To estimate the consequence of varying count dispersion on downstream statistical analysis, we conducted power and sample size calculations using these common dispersion estimates (Fig. 5E; Fig. S3; Table S5). When dispersion was estimated from *n* = 3 tumours, the estimates of the sample size required to achieve 0.8 statistical power had a greater range and lower median, with the minimum across simulations at n = 3 being approximately 1.5- to 2-fold less than that of the most conservative estimates (maximum across simulations) with *n* = 10/*n* = 20 tumours (except where the estimated sample sizes required were themselves close to *n* ≤ 3). This suggests that when the sample size is estimated from a smaller pilot dataset, a conservative sample size inflating factor of 2 is needed to ensure the correct level of statistical power in a full dataset. When using the more precise *n* = 10 dispersion estimates for small tumours, our simulations showed that at a log_2_ effect size of 2, a median estimate of 35 tumours (range 34–37 across simulated datasets) is required to achieve power of 0.8 with 100,000 gRNAs, progressively reducing to 3 tumours (range: 3–4) with 2000 gRNAs. We estimate that there would be 170 clones/gRNA when using a library of 2000 gRNAs. For large tumours (dispersion estimates from *n* = 20 tumours) and 4000–100,000 gRNAs, >50 tumours per group were required to achieve power of 0.8 (Fig. 5E; Fig. S3A; Table S5) at effect size of 2, falling to 20–50 tumours at 500–2000 gRNAs, and <20 tumours at library sizes of <500 gRNAs. Although our power calculations indicate very small libraries are needed to achieve good power with feasible group sizes (<20–30) at this effect size, these can also be interpreted in the context of the number of clones present: we estimate ~100–400 clones/gRNA will survive in libraries of <500 gRNAs in large tumours. These estimates highlight how the reductions in count dispersion, achieved through the use of a smaller gRNA library, substantially reduce the sample sizes needed to achieve good statistical power.

## Discussion

In vivo CRISPR/Cas9 screens offer the opportunity to identify genes responsible for a specific phenotype in response to selective pressure, while maintaining the complex multicellular interactions of the tumour microenvironment. In this study, we investigated several parameters that could be optimised when conducting these screens, including cell line, host mouse strain, tumour collection time and gRNA library size. Our low TD_50_ HCT116/54C line retained >99% gRNA representation in cultured cells and had much higher gRNA representation than the moderate TD_50_ cell line UT-SCC-74B when GeCKO-transduced cells were inoculated into NSG mice at 10^7^ cells. Although not definitive since only two models were used, our data does suggest that using a conducive mouse host and cell line with a low TD_50_ and, therefore, potentially a high frequency of tumour-initiating cells can lead to high gRNA representation on tumour initiation.

Despite the high gRNA representation at tumour initiation with our HCT116/54C GeCKO tumour model, a considerable loss in gRNA representation was observed as tumours were grown to >1 cm^3^. The loss of representation was accompanied by a substantial increase in read count inequality that was highly variable between tumours and observed with both targeting and non-targeting gRNA, suggesting that stochastic differences in clonal growth and survival rather than gRNA-induced selection were the dominant driver. These clonal dynamics would include both random clonal expansion (where some clones have a growth advantage) and random clonal dropout (where some clones have a survival disadvantage). Our data suggests, however, that it is stochastic clonal expansion, likely expedited by the genetic bottleneck of tumour initiation [46], that is a more important limiting factor for in vivo CRISPR/Cas9 screens. In the unequal read count distributions of the tumour samples, the majority of reads were from only a small fraction of gRNAs, with up to 1.4–17% of all reads from a single gRNA in the large tumours, probably from a single expanded clone. The random expansion of individual clones has also been reported as a confounding factor in cell-based CRISPR/Cas9 screens [47] and is common in xenograft models [46, 48]. Clonal expansion can also lead to apparent dropout due to sampling effects (sampling zeroes; ref. [43]), where clones that remain present in the population but are less abundant become difficult to detect due to repeated sampling of the expanded clones.

Although there were some indications of gRNA-driven selection among our tumour samples, ultimately, random clonal dynamics within the tumours prevented us from identifying drug sensitivity genes in our study, due to the high count dispersion. Indeed, the BCV estimates we observed of about 1 for small tumours and approaching 3 for large tumours vastly exceed typical values observed in count data from RNAseq [49]. Given the high dispersion, we ran simulations to identify the parameters our model would require to have 80% statistical power to detect gRNA-mediated gene selection. Our simulated datasets indicated that count variance could be reduced by reducing library size and increasing averaging across clones, increasing statistical power to a level where gRNA-driven selection (log_2_ fold-change 2) is detectable in a study of feasible size with 20,000 or fewer gRNAs. In large tumours, however, our simulations suggested very small library sizes of ≤500 gRNAs are needed to achieve similar power at the same effect size when large groups (*n* ~ 20–30) are used. This suggests that the improvements made by using a smaller library are rapidly diminished by cumulative time/growth-dependent stochastic effects. To overcome this strong time-dependent effect, initiating tumour treatment when tumours are small (~150 mm^3^) and collecting tumours 7–14 days after treatment when tumours at most are moderate in size (<~750 mm^3^) may allow count variance to be adequately constrained to retain sufficient statistical power to detect smaller treatment effects. When assessed in terms of the number of clones/gRNA, our simulations suggest a rule of thumb when choosing a library size is coverage in the range of 50–400 clones/gRNA, which is similar to the suggested coverage of 25–250 cells/gRNA (100–1000 cells/gene) for in vitro screens [45, 50], although expressed in terms of clones instead of cells. In terms of likely effect sizes, the mean estimated level of negative selection (for essential genes) in our large tumours was low (−0.39 log_2_ fold-change) but somewhat greater than in vitro samples cultured over a similar period of 40 days (−0.19 log_2_ fold-change; Supplemental Results; Fig. S8), suggesting a similar magnitude of selection would be observed in in vivo screens as that observed in in vitro screens when coverage is sufficient, and the screens are adequately powered. Although this estimate was lower than the effect sizes used in our power calculations, it represents an average value rather than the effect size that would occur with the most selective genes and the most active gRNAs. In addition, this number likely reflects the performance of the GeCKOv2 library, which is known to be poorer than more recent libraries [44], and is not suitable to be generalised across gRNA libraries. The use of gRNAs with high targeting efficiency [44, 51] is likely to be important to maximise the effect sizes and ensure success in in vivo screening.

A number of successful transplantable in vivo CRISPR/Cas9 screens have been reported in the literature. Consistent with our simulations of the smaller datasets, most of these used smaller libraries limited to a subset of genes consisting of <10,000 gRNAs, with libraries potentially further subpooled [52–55]. In a non-exhaustive survey of the literature for previous whole genome in vivo screens, we found that, when sufficient detail was provided, most datasets resembled that of the current study in terms of loss of representation, read count inequality and highly expanded counts of a small number of clones, particularly in larger tumours [28, 29, 31, 32, 56–58]. Two studies, however, report considerably higher gRNA representation and lower count inequality than our model [30, 33]. In both of these studies, tumours were inoculated at 3 × 10^7^ cells into mice, a 3-fold increase on our inoculum. This extends to a ~6-fold increase in cells/gRNA (~450 vs. ~84 in our study) as they used the GeCKOv2A half-library, and this may have contributed to greater averaging across clones in tumours and a reduction in count variance compared to our study. However, it is likely other variables, such as improved tumour growth characteristics due to the particular cell line/host combination used (i.e. more tumour-initiating cells), also contributed to the significantly better-quality data compared to all the other long-term whole genome in vivo screens. Indeed, in our own data, a considerable difference in performance was observed between the HCT116/54C GeCKO and UT-SCC-74B GeCKO models, with the use of NSG over NIH-III hosts providing only a minor improvement.

Our study provides several insights regarding the underlying cell growth characteristics in in vivo tumour xenograft screens with implications for the analyses of the resulting data. Firstly, we show that count dispersion is dependent on sample type (cells versus tumours) and increases with growth time in both cell and tumour samples. These observations have an intuitive explanation—count dispersion is driven by random clonal dynamics, and the extent of this effect is expected to be cumulative over time and greater in tumours due to the founder effect and stronger microenvironmental differences between clones. Although not unexpected, we are not aware that this issue has been highlighted previously. We also show that the variable nature of count dispersion across samples poses a risk of improperly controlling statistical error when samples of mixed dispersion are combined (e.g. small and large tumours, cells cultured from significantly different periods). Methods developed to analyse sequencing count data, including methods originally developed for RNAseq such as edgeR [37, 49] and the negative binomial method for gRNA counts in MAGeCK [38], typically allow for heterogeneity in variance among different genes/gRNAs by modelling a mean-variance trend but do not allow for heteroskedasticity among sample groups. While this may not be an issue in RNAseq data where counts measure gene expression, we believe it warrants further attention in analyses of CRISPR/Cas9-screen gRNA data, given random clonal dynamics are a potentially large source of heteroskedasticity between sample groups, even for purely in vitro screens. As a minimum, care should be taken when analysing these data to ensure all groups have similar variances. If required, tools that separate the estimation of the dispersion from statistical tests, such as edgeR, could be used to perform conservative statistical analyses where dispersion is estimated from the group(s) with the most variance rather than across all groups. In addition, the use of linear model-based voom/limma analysis with sample-specific weights [59] may improve analyses by modelling sample-specific variation; we did not test this method in the present study as our data contained a high proportion of zero counts, which we considered to be unsuitable for linear model analysis of counts. In addition, the characteristics of our data could be used to parameterise computational tools [50] that model the clonal dynamics in in vivo screens, allowing more sophisticated in silico simulations to be carried out to assess the contribution of various screen parameters and ensure studies are adequately powered.

Finally, our study provides a pathway towards a more rational approach in designing in vivo CRISPR/Cas9 screens for drug sensitivity genes. The first step in designing a screen is to consider the trade-offs between library size and resulting statistical power. As we have shown, smaller libraries can have greater power and are ideal if a focussed set of genes is being investigated, however may not be suitable for more exploratory research. A two-stage screening process with candidates for in vivo screening first identified within in vitro screens in simple cultures or more biologically complex 3D spheroid or organoid cultures [60, 61] could be used if there is no candidate-focused gene set initially available. If a full genome library in vivo is required, apart from more recent optimised whole-genome libraries with higher targeting efficiency (e.g. Brunello or Brie) [44], a further optimised “minimal” library (~38,000 gRNAs) could be used to provide some reduction in library size [51]. Once the initial candidate strategy is chosen, it may be beneficial to test multiple cell lines and animal hosts to identify the combination that provides the best tumour growth characteristics in pilot tumour growth experiments (*n* = 3 tumours), similar to those performed in the current study. The resulting gRNA counts should be evaluated not only in terms of representation but also count inequality and variance, with the combination with the lowest variance preferred to increase the chances of detecting gRNA-driven selection. It may also be useful to evaluate different tumour collection timepoints to ascertain the degree to which count variance increases over time. Regardless, a pilot study (*n* = 3) of the chosen cell line and host should be conducted at the candidate tumour collection timepoint (not done in the current study) prior to commencing a full-sized experiment. Power calculations can then be performed using these pilot data to estimate the sample size needed to detect treatment effects at the gRNA level. Here, it is worth noting that our simulated datasets suggest a sample size inflating factor of 2 may be needed to provide a conservative estimate due to imprecise variance estimates from limited data (an expected issue when using pilot data to estimate power for a main experiment; ref. [62]). If the estimated sample size proves infeasible, it would be preferable to make changes to the screen design to improve power (library size, cell line, host) rather than proceeding with an underpowered in vivo screen, for both resource and ethical reasons.

In summary, we report that the main limiting factor for in vivo CRISPR/Cas9 screens in detecting gRNA-driven changes among treatment groups is variable clonal dynamics within tumours leading to high count variance. This can be addressed to a degree by cell line and mouse host choice. We found that count variance increased with increasing growth time, likely due to random differences in cell growth and survival in the tumours. In large tumours of our model, count dispersion accumulated to a very high level, with the populations dominated by relatively few individual clones. Using simulated binned datasets, we showed that count dispersion can be reduced by the use of small tumours (or those grown for a short period of time) and greater averaging using smaller gRNA libraries. Based on these analyses, we conclude that our xenograft model would have been suitable for short-medium duration in vivo screens using a more focused library of up to several thousand gRNAs. These findings will likely extend to other xenograft models with similar in vivo growth characteristics.

### Supplementary information

Supplementary Information

Table S1

Table S2

Table S3

Table S4

Table S5

Supplementary Data File 1

Supplementary Data File 2

Supplementary Data File 3

Supplementary Data File 4

## Data Availability

The gRNA count dataset, analysis code and other associated files are available in Figshare (10.17608/k6.auckland.23037053).
