# Supplementary material for: Clonal dynamics limits detection of selection in tumour xenograft CRISPR/Cas9 screens
Source: Cancer Gene Ther. 2023 Sep 8;30(12):1610–23. doi: 10.1038/s41417-023-00664-5 (PMC10721547; doi:10.1038/s41417-023-00664-5)
Supplement: Supplementary file 1 — Supplementary Information [file 41417_2023_664_MOESM1_ESM.pdf]

Clonal dynamics limits detection of selection in tumour xenograft CRISPR/Cas9 screens

Tet Woo Lee[1,2*], Francis W. Hunter[1,2,3], Peter Tsai[2,4], Cristin G. Print[2,4], William R. Wilson[1,2], Stephen M.F. Jamieson[1,2,5*]

[1]Auckland Cancer Society Research Centre, University of Auckland, Auckland, New Zealand.

[2]Maurice Wilkins Centre for Molecular Biodiscovery, University of Auckland, Auckland, New Zealand.

[3]Oncology Therapeutic Area, Janssen Research and Development, Spring House, PA, USA

[4]Department of Molecular Medicine and Pathology, University of Auckland.

[5]Department of Pharmacology and Clinical Pharmacology, University of Auckland, Auckland, New Zealand.

Correspondence to: Tet Woo Lee (tw.lee@auckland.ac.nz) or Stephen Jamieson (s.jamieson@auckland.ac.nz)

Supplementary Information:

Supplementary Data Files: p3

Supplementary Figures S1-S10: p4-17

Supplementary Tables S1-S5: p18

Supplementary Methods: p19-26

Supplementary Results: p27-30

References: p31

Supplementary Data Files

Supplementary Data File 1

edgeR_exacttest_CvsB_grna.tsv: gRNA-level comparison for group C (6-thioguanine) vs group B (control) using edgeR exactTest in tab-separated values format. Description of each column in the first row of the file.

Supplementary Data File 2

alphaRRA_CvsB_gene.tsv: gene-level comparison for group C (6-thioguanine) vs group B (control) using alpha-RRA on edgeR exactTest results with alpha set to an edgeR FDR cutoff of 0.2 in tab-separated values format; positive and negative selection combined. Description of each column in the first row of the file.

Supplementary Data File 3

edgeR_exacttest_DvsB_grna.tsv: gRNA-level comparison for group D (evofosfamide) vs group B (control) using edgeR exactTest in tab-separated values format. Description of each column in the first row of the file.

Supplementary Data File 4

alphaRRA_DvsB_gene.tsv: gene-level comparison for group D (evofosfamide) vs group B (control) using alpha-RRA on edgeR exactTest results with alpha set to an edgeR FDR cutoff of 0.2 in tab-separated values format; positive and negative selection combined. Description of each column in the first row of the file.

Supplementary Figures

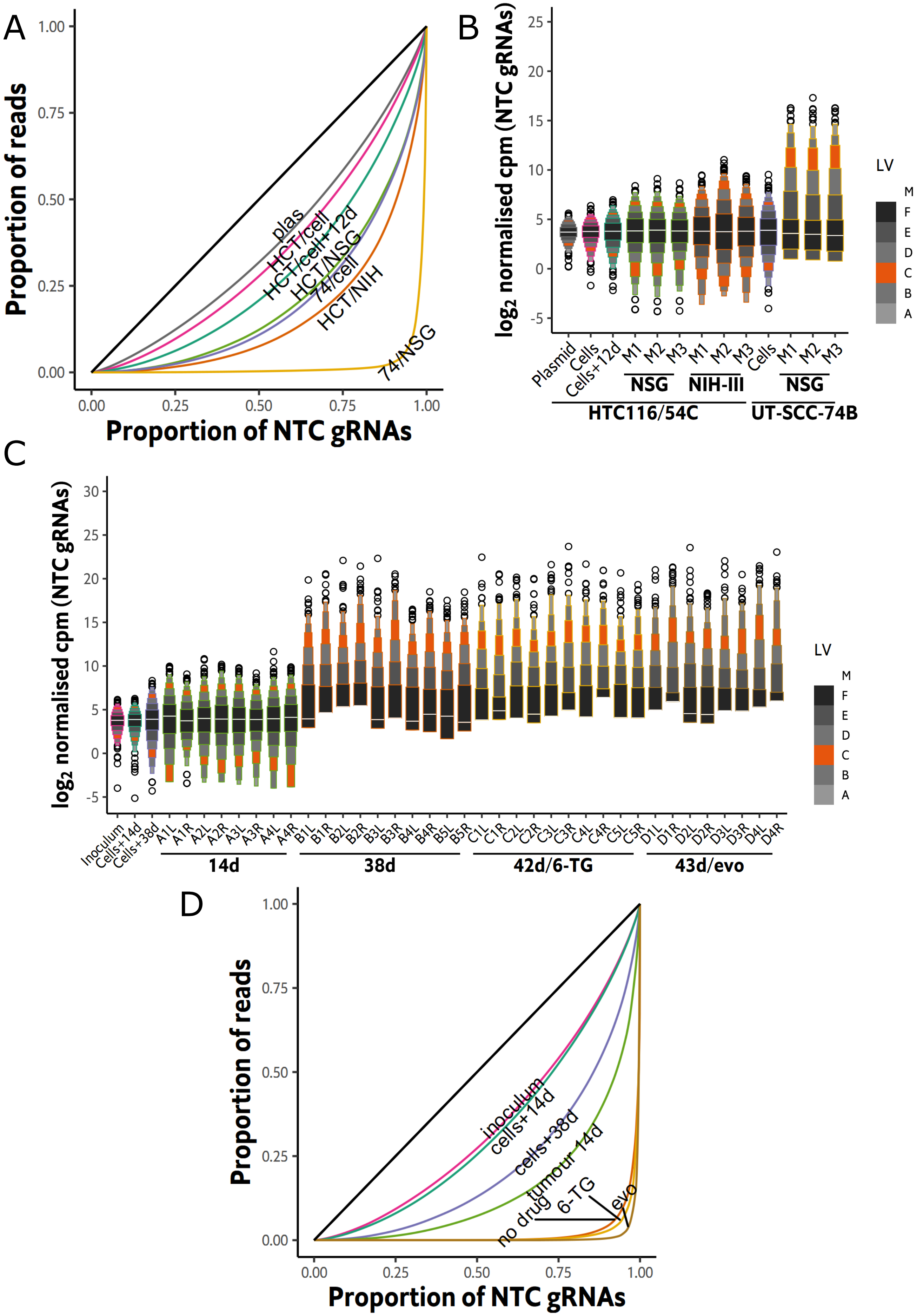

Figure S1: (A) Lorenz curves to show distribution of NTC gRNA read counts from the pilot study (plas: plasmid; HCT: HCT116/54C; 74: UT-SCC-74B; NIH: NIH-III). (B) Letter-value plot of log normalised counts per million (log-ncpm) for NTC gRNAs in plasmid, cell inocula and GeCKO tumour samples from the pilot study. Median log_2 normalised cpm values for plasmid was 3.71, HCT116/54C GeCKO cells 3.72-3.73, HCT116/54C GeCKO NSG tumours 3.71-3.74, NIH-III tumours 3.68-3.76, UT-SCC-74B GeCKO cells 3.78, UT-SCC-74B GeCKO tumours 3.63-4.01. (C) Letter-value plot of log normalised counts per million (log-ncpm) for NTC gRNAs in cell inocula and GeCKO tumour samples from the larger study (6-TG: 6-thioguanine; evo: evofosfamide). (D) Lorenz curves to show distribution of NTC gRNA read counts from the larger study. For the Lorenz plots, the curve for summed PCR replicates (plasmid and cell samples) or median tumour in a group is highlighted. The black line is the line of equality.

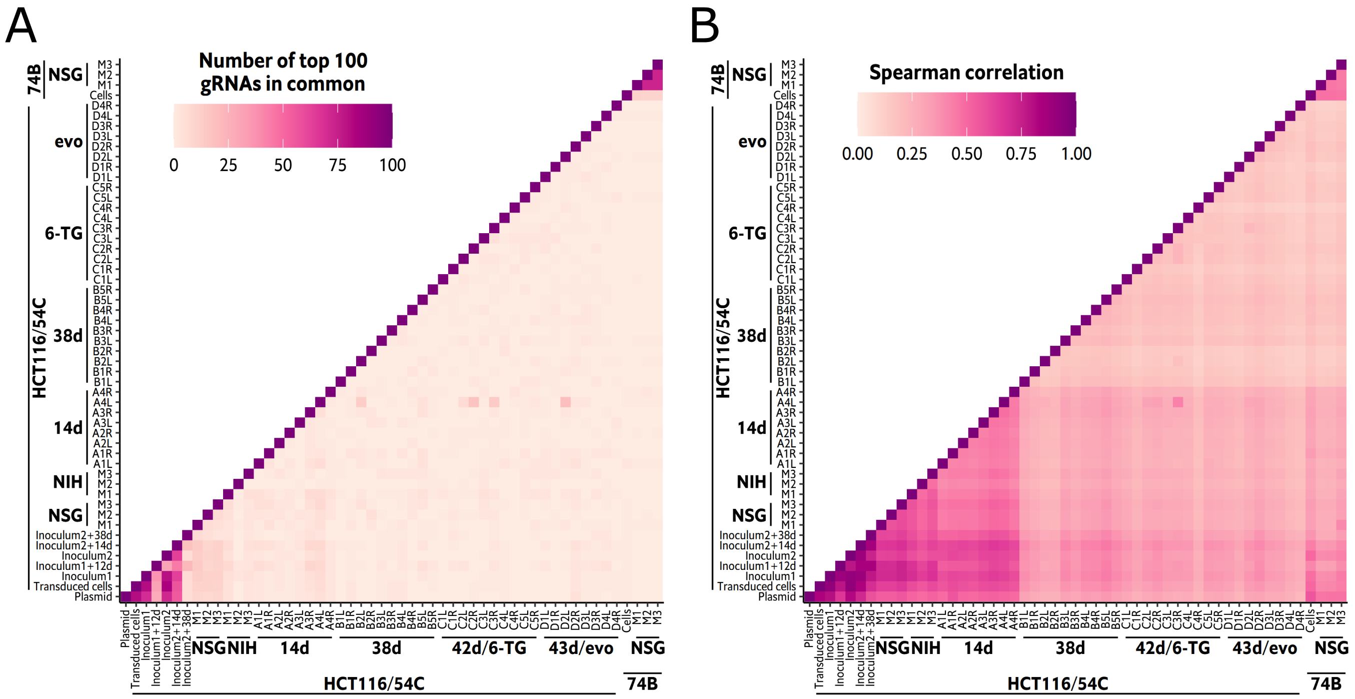

Figure S2: (A) Heat map showing the number of the top 100 gRNAs with the highest counts that are shared among each pair of samples. The high number (dark magenta) indicates that the top gRNAs in each pair of samples are similar, while a low number (pale orange) indicates each pair of samples has different top gRNAs. (B) Heat map showing the matrix of Spearman correlation coefficients between each pair of samples with a darker colour indicating greater correlation.

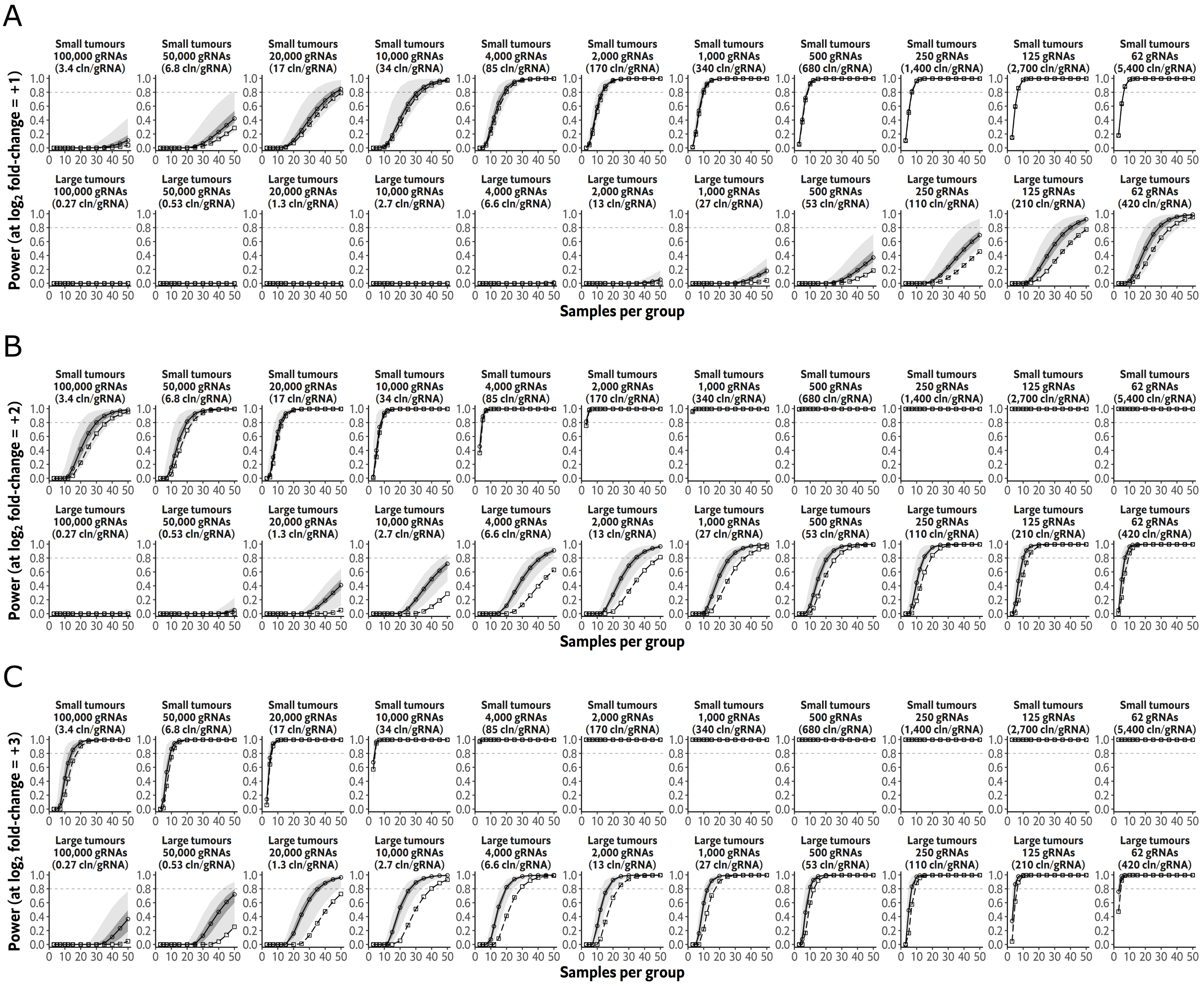

Figure S3: Power curves determined from the common dispersion estimates of simulated datasets of various library sizes at a $\log_2$ effect size of (A) 2, (B) 1 and (C) 3 for small and large tumours. Results from all simulated library sizes have been included. cln/gRNA: clones/gRNA.

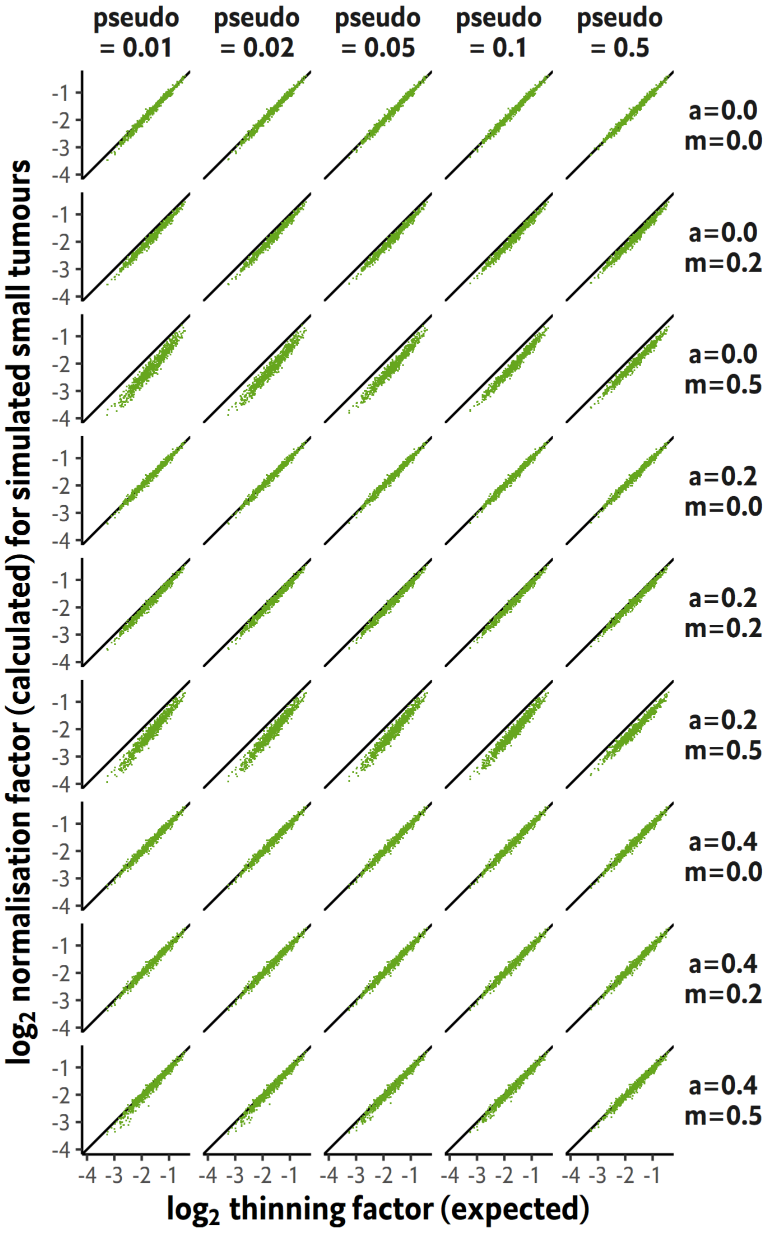

Figure S4: Plots of calculated MPM normalisation factors against expected values in simulated small tumour samples, for a variety of parameters. Each point represents the normalisation factor for a single sample simulated using binomial thinning (n=8 samples × 100 datasets). Points are plotted on a log scale and the line of equality (calculated=expected) is shown in black; a correctly calculated

normalisation factor should be equal to the expected values and lie on top of this line. Normalisation factors were calculated in combined datasets of 8 simulated small tumours, three groups of simulated large tumours (28 in total), and the comparator plasmid sample and 9 samples of gRNA counts from HCT116/54C cells.

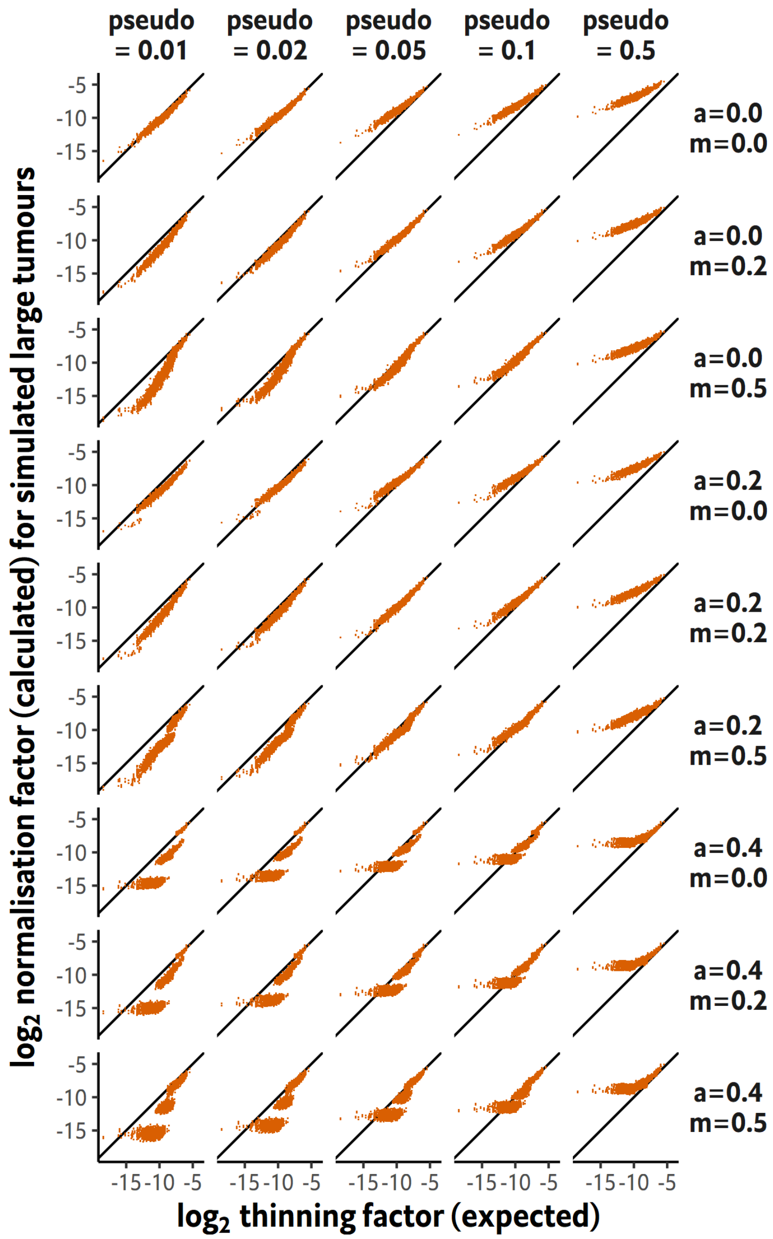

Figure S5: Plots of calculated MPM normalisation factors against expected values in simulated large tumour samples, for a variety of parameters. Each point represents the normalisation factor for a single sample simulated using binomial thinning (n=28 samples × 100 datasets). Points are plotted on a log scale and the line of equality (calculated=expected) is shown in black. Normalisation

factors were calculated in combined datasets of 8 simulated small tumours, three groups of simulated large tumours (with different systematic effects), and the comparator plasmid sample and 9 samples of gRNA counts from HCT116/54C cells.

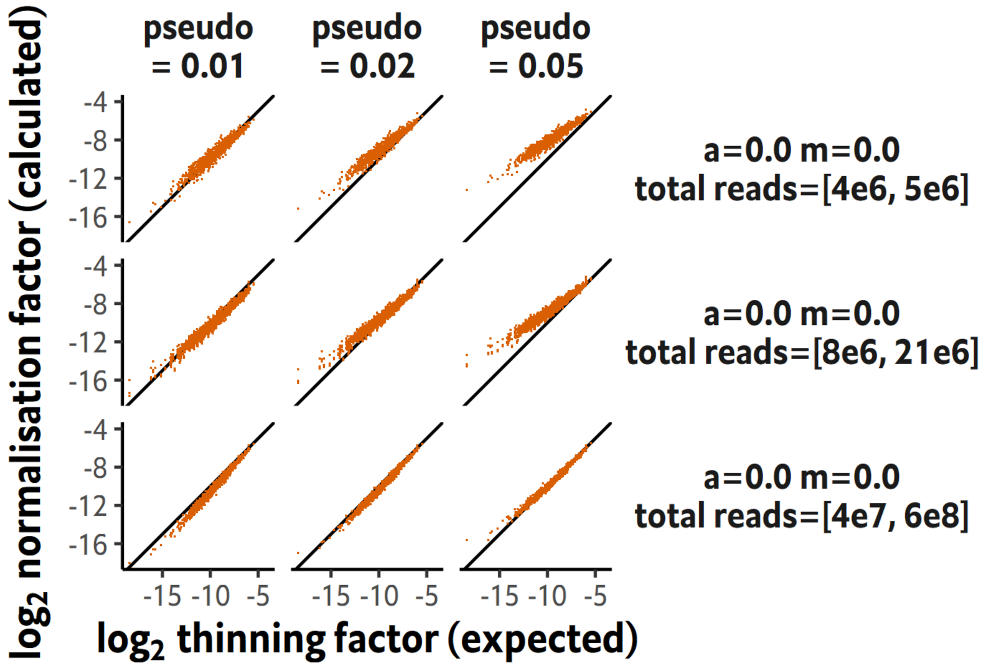

Figure S6: Plots of calculated MPM normalisation factors against expected values in simulated large tumour samples with a different number of total reads, for three pseudocount parameters. Each point represents the normalisation factor for a single sample simulated using binomial thinning (n=10 samples × 100 datasets). Points are plotted on a log scale and the line of equality (calculated=expected) is shown in black. Normalisation factors were calculated from datasets of 10 simulated large tumours (with different systematic effects) and the comparator plasmid sample.

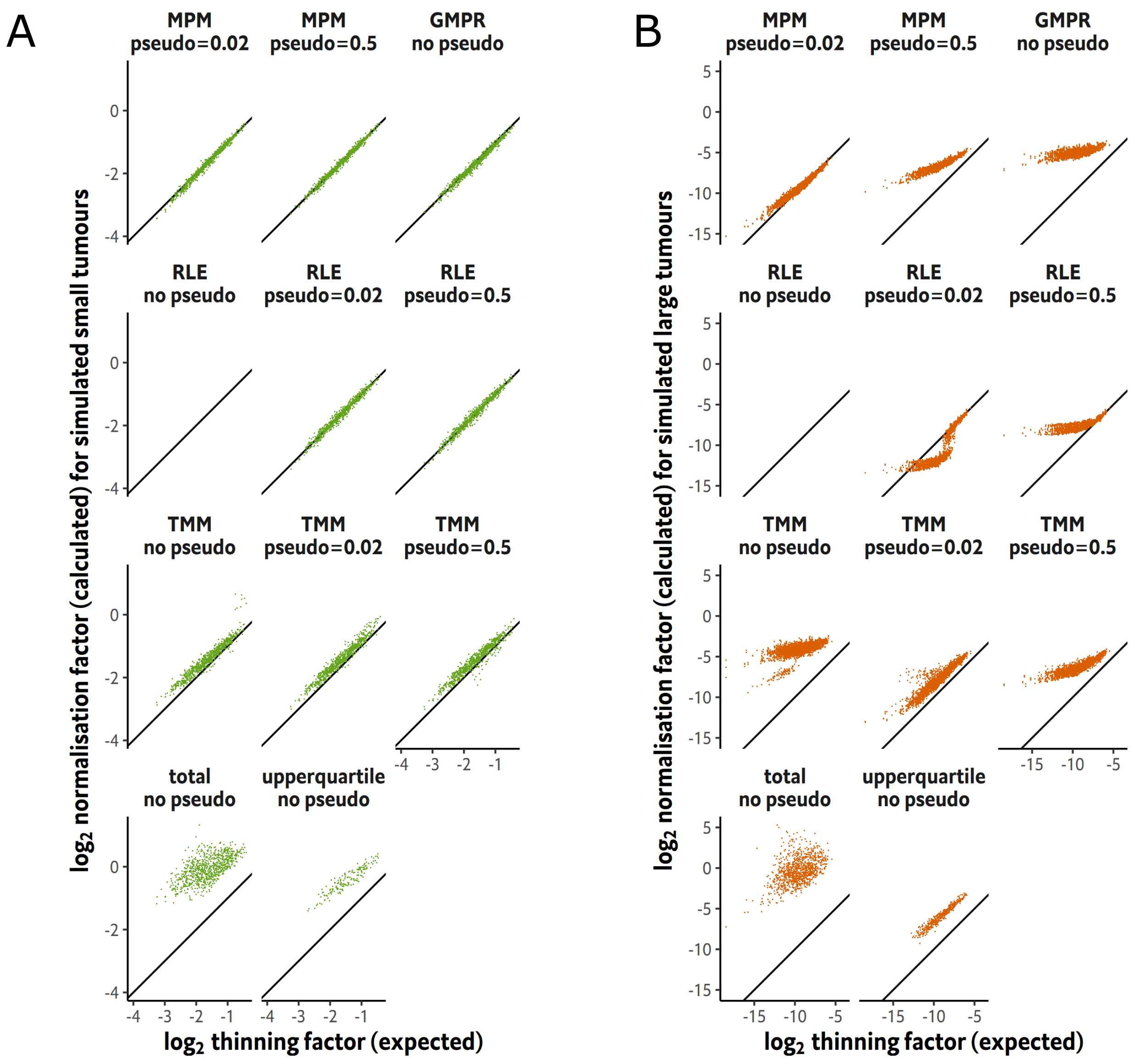

Figure S7: Plots of calculated normalisation factors against expected values in simulated (A) small or (B) large tumour samples for a variety of normalisation methods. Each point represents the normalisation factor for a single sample simulated using binomial thinning (A: n=8 samples × 100 datasets; B: n=28 samples × 100 datasets). Points are plotted on a log scale and the line of equality (calculated=expected) is shown in black. TMM, RLE and GMPR were used with the default parameters; if indicated, zero counts were set to a

pseudocount prior to the calculations. The RLE method with no pseudocount failed in all datasets due to presence of too many zeroes, while the upper quartile method failed frequently when the upper quartile was 0. All normalisation methods were performed using the (simulated) NTC gRNA counts only, but corrected for the total (all gRNA) library size. Normalisation factors were calculated from combined datasets of simulated small and large tumours, the comparator plasmid sample, and 9 HCT116/54C cell counts and 9 samples of gRNA counts from HCT116/54C cells.

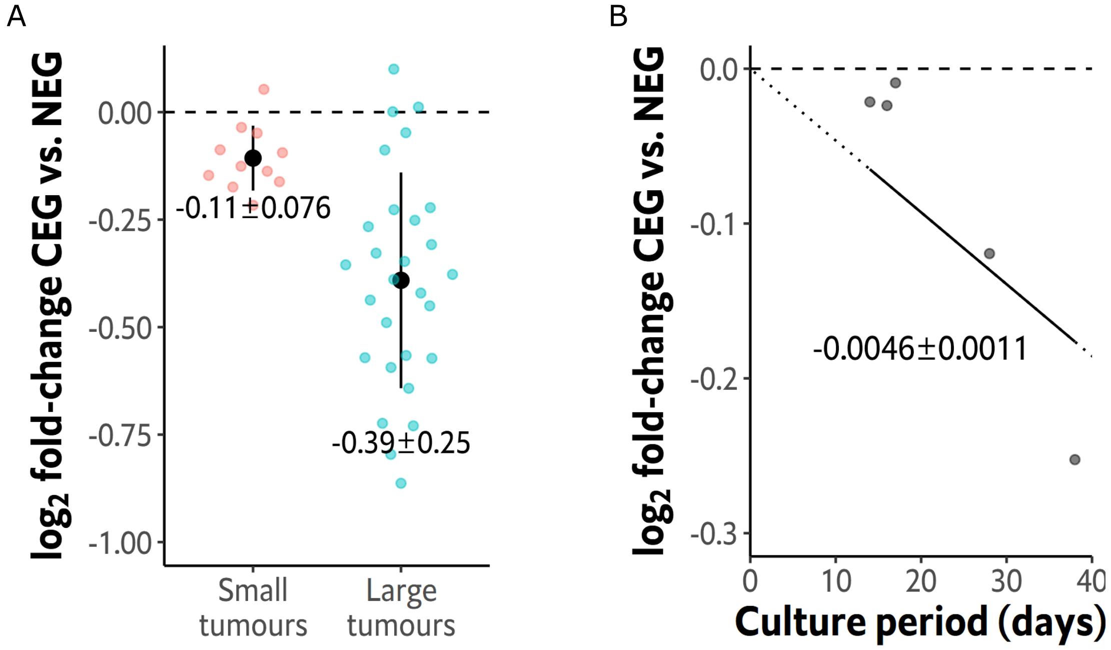

Figure S8: Plots of $\log_2$ fold-change of common essential genes (CEG) compared to non-essential genes (NEG) in HCT116/54C GeCKO (A) tumours and (B) in vitro samples as an estimate of the level of CEG gRNA depletion (negative selection). Each point represents the mean across 100 binned/subsampled datasets for a single tumour or cell sample, and is corrected for the level of CEG depletion in the corresponding parent sample. In (A), the mean and standard deviation across samples is shown. In (B) a slope-only linear regression has been fit with the label indicating the fitted slope and its standard error (in $\log_2$ fold-change per day); for a 40-day period the level of depletion would be −0.19±0.045.

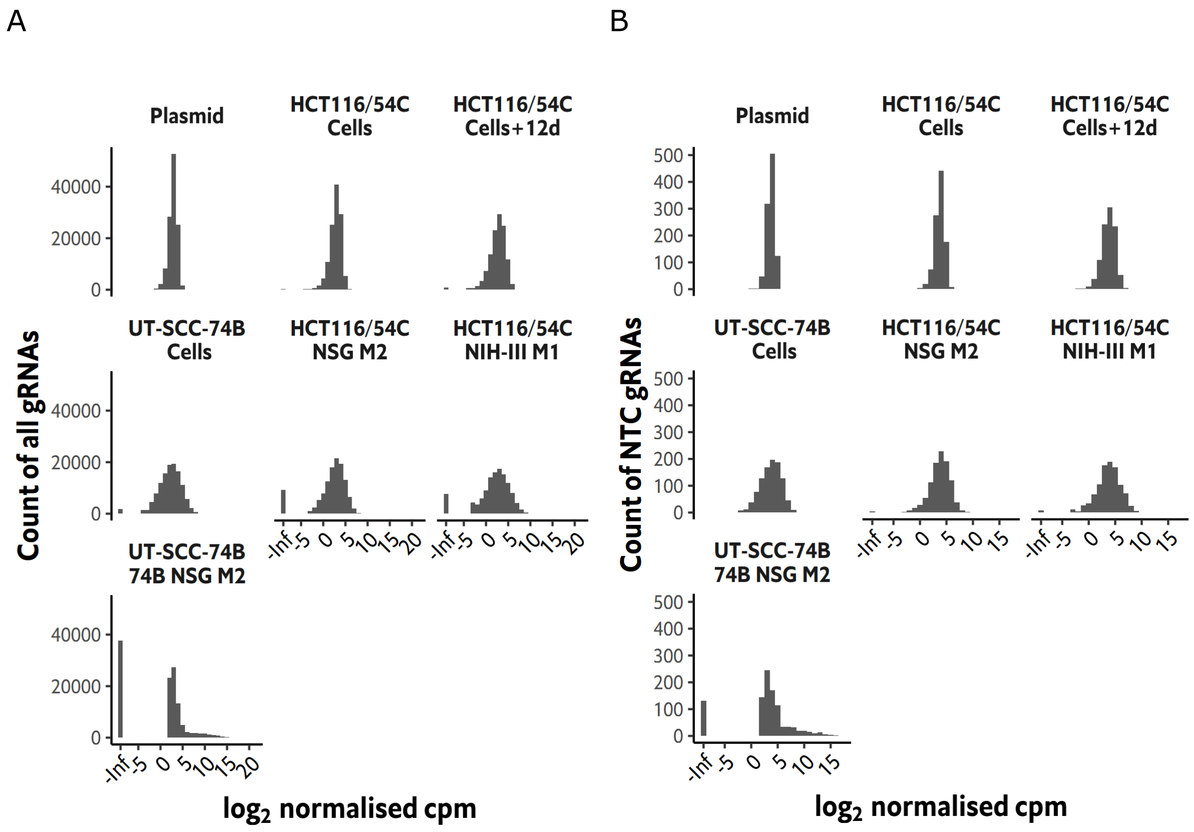

Figure S9: Histograms of normalised $\log_2$ counts per million for representative samples in the pilot study. (A) shows the histogram for all gRNAs and (B) for NTC counts only. Each bin is one $\log_2$ counts per million unit in size; zeroes are plotted at -Inf. For plasmid/cell samples, PCR replicates were summed prior to plotting. For tumour samples, the sample with the median 90th percentile count in its group was selected to be included in the histogram.

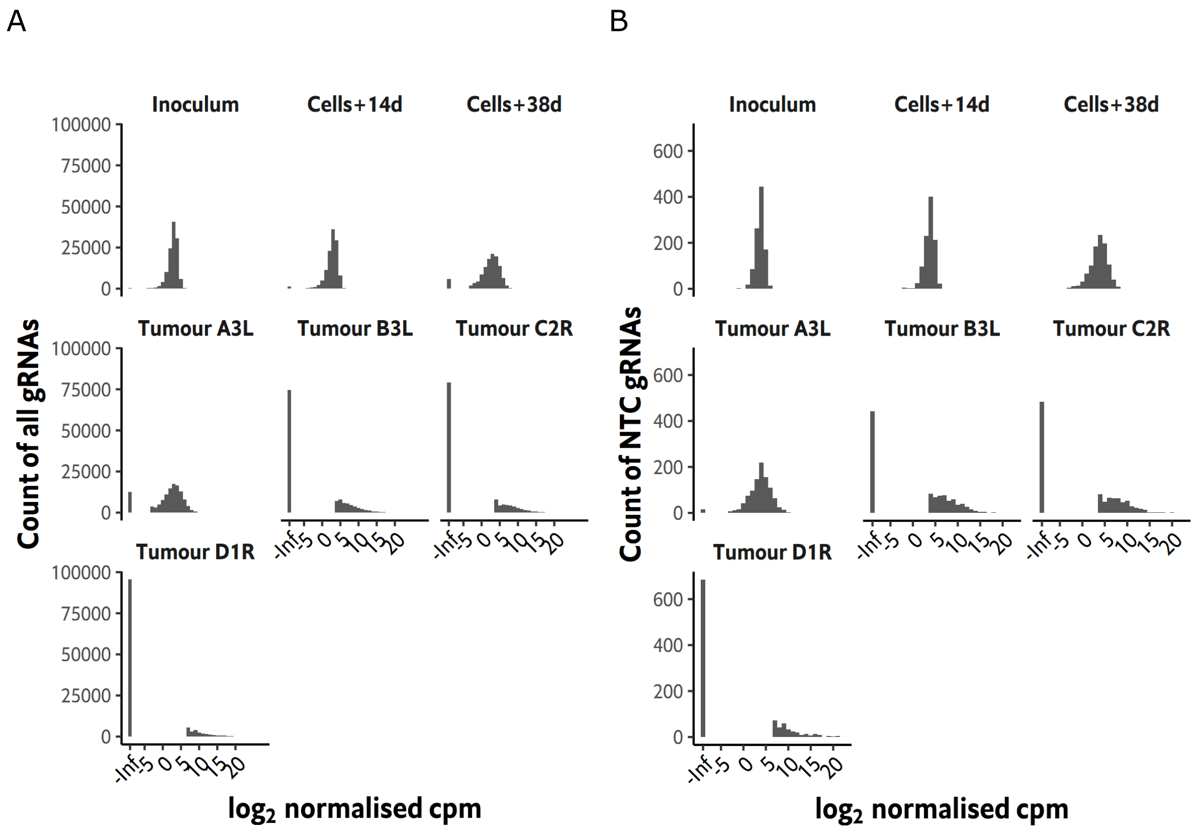

Figure S10: Histograms of normalised log_2 counts per million for representative samples in the pilot study. (A) shows the histogram for all gRNAs and (B) for NTC counts only. Each bin is one log_2 counts per million unit in size; zeroes are plotted at -Inf. For plasmid/cell samples, PCR replicates were summed prior to plotting. For tumour samples, the sample with the median 90th percentile count in its group was selected to be included in the histogram.

Supplementary Tables

The following supplementary tables can be found in the supplementary excel file:

Table S1 Details of all gRNA reads for samples in the pilot study

Table S2 Details of NTC gRNA reads for samples in the pilot study

Table S3 Details of all gRNA reads for samples in the larger study

Table S4 Details of NTC gRNA reads for samples in the larger study

Table S5 Sample size estimates at power 0.8 in simulated datasets with varying numbers of gRNAs

Supplementary methods

Log counts per million

Given a dataset of gRNA read counts r_{gi} for samples $i = 1,...,N$ and gRNAs $g = 1,...,G$, log counts per million (log-cpm) is defined:

$$\mathrm{Y_{gi}} = \log_2\left(\frac{\mathrm{r_{gi}}}{\mathrm{R_i}} \times 10^6\right)$$

where R_i is the total number of counts for the sample (library size). The log-cpm is a measure of the proportion of counts in a sample that are for a given gRNA but is a poor measure of relative changes in the count for the gRNA across samples since the denominator (R_i) is strongly affected by disproportionately large counts of certain gRNAs. To account for this, we calculated log normalised counts per million (log-ncpm), by replacing R_i with S_i, the normalised library size for the given sample (see below). In the above definition, Y_{gi} is not defined when the read count is zero; in such cases zero counts can be set to a predefined pseudocount (c_{pseudo}) when it is necessary that all log-cpm values are defined. In the current study, pseudocounts were only used when calculating normalisation factors.

Read count normalisation

We devised a novel normalisation method, which we call MPM (mean of pairwise M values), to normalise our read count data by calculating normalised library sizes. This method is similar to the TMM [1] and RLE [2] methods commonly used for RNAseq data, and GMPR [3] that was developed to deal with zero inflated counts in microbiome sequencing data. The general principle of these methods is that there are expected to be non-differential features (e.g.

expressed genes or gRNAs) across samples. The non-differential features will be in the centre of the distribution of count ratios. Relative normalisation factors between samples can be robustly estimated from the central non-differential features using a median or trimmed mean of count ratios.

In the MPM method, we defined two possible stages of clipping or trimming outlying counts and ratios when calculating normalisation factors. First, the influence of outlying high counts can be reduced by clipping the top and bottom *a%* of counts in each sample – at each end, counts above or below the *a*-th percentile are replaced with the count at the *a*-th percentile at that end. The motivation is this step is to reduce the influence of large outlying counts and is analogous to 'A' trimming in the TMM method, except all counts are retained through the use of clipping, as opposed to trimming, each end. Although trimming is performed at each end, for highly zero-inflated data, trimming at the lower end often has no effect as the lower *a*-th count is also zero.

The pairwise log-fold-change (M) between samples *i* and *j* is then calculated across gRNAs from *a*-clipped counts r'_{gi}, with *m*% of valid ratios discarded from each end to calculate the trimmed mean:

$$M_{ij} = \underset{g \in \{1,\dots,G\} \mid r_{gi} + r_{gj} \neq 0}{\text{trimmed mean}} \left(log_2 \frac{r''_{gi}/R_i}{r''_{gj}/R_j} \right);\; r'' = \begin{cases} c_{pseudo} \text{ when } r' = 0 \\ r' \text{ when } r' > 0 \end{cases}$$

This is analogous to 'M' trimming in the TMM method. The maximum of *m*=0.5 uses the median *M* value. Note that only gRNAs that have zero counts (before *a*-clipping) in both samples are excluded from this calculation; gRNAs with a zero count in one sample are retained with their *a*-clipped counts given a pseudocount of c_{pseudo} to avoid taking the log of zero. Although the use of a pseudo-count for zeroes has some weaknesses [3], zero counts are informative

in terms of the ordering of two samples – a zero count in one sample is an indication the count is lower in the sample with a zero than the sample without a zero. Excluding all zero counts thus biases the count ratio upwards for samples with many zeroes. Therefore, we prefer to retain zero counts when calculating normalisation factors. The use of small pseudocounts is also consistent with the approximation of zeros as sampling zeros which have a finite but small actual abundance [4]. The gRNAs that have zero counts in both samples provide no information about the ordering or ratio of the two samples, and is better to exclude these from the calculation as the tendency would otherwise be to bias the count ratio towards 1 (c_{pseudo}:c_{pseudo}).

The normalisation factor s_i for sample *i* on a log scale is then calculated as the mean of *M* values to all other samples:

$$log_2 s_i = \left(\frac{1}{N-1}\sum_{j=1,j\neq i}^{N} M_{ij}\right) - \left(\frac{1}{N-1}\sum_{j=1,j\neq k}^{N} M_{kj}\right)$$

The second term scales the normalisation factors to be relative to arbitrary sample *k* (i.e. s_k = 1). Note in MPM, normalisation factors are estimated from all pairwise ratios, as in GMPR. MPM with *a*=0, and *m*=0.5 is similar to GMPR except ratios where only one count is zero are retained in the calculation by setting the zero to a pseudocount; GMPR does not use pseudocounts and excludes all zeroes. Therefore, MPM can be seen as a generalisation of the GMPR method with additional trimming options and the use of a pseudocount. The normalisation factor is a correction factor for library sizes, with normalised library size calculated as:

$$S_i = \mathrm{R_i} s_i$$

When normalised library sizes are used in place of unnormalised library sizes, log-ncpm becomes a measure of relative counts across samples. As the gRNA library contains 1000 non-targeting controls (NTCs), which can be assumed to be neutral, and thus non-differential on average, the size factors can be calculated using only NTC gRNAs i.e. $g \in \{NTC\ gRNAs\}$ to give log-ncpm normalised to neutral gRNAs; note that full library size *R* is used in all calculations even when r'_g is subsetted to NTC gRNAs.

Binomial thinning simulations

To compare normalisation methods, we bootstrapped (sampled with replacement) the gRNA counts from the plasmid sample to a total of 1.6×10^{13} reads. This bootstrapped data contained a median count 1.2×10^{8} per gRNA, necessary to allow individual gRNA counts of up to several million following binomial thinning.

Next, we used binomial thinning using the *seqgendiff* package in R [5] to generate simulated datasets each containing 10 thinned columns (samples) mimicking small tumour and large tumour samples. In each dataset, a random signal was applied to expand/reduce each count to account for stochastic clonal expansion/reduction, while fixed signals were applied across each row to all columns in a dataset to account for systematic differences in all samples due to underlying cell fitness in NTC gRNAs and guide-specific effects with a general dropout effect. The effect sizes of the fixed signals were varied slightly for each column. The random and fixed signals were randomly sampled from a normal distribution with parameters given in Table S6; separate parameters were used to simulate small tumours and large tumours. In addition, each column was

further uniformly thinned to give a certain total number of reads. This uniform thinning causes many of the low counts to drop to zero and effectively zero-inflates the counts. These simulated datasets resembled the counts from our large and small tumour samples in terms of gRNA total counts, representation and count inequality. The key assumption being made in these simulations is that the combined signal applied to NTC gRNAs has a mean of 0 despite potentially large standard deviation that causes outlying high count values accompanied by many zeroes.

Table S6 Parameters used for binomial thinning simulations to compare normalisation methods. Where parameters are given as ranges, a value was sampled from a uniform distribution within the range for each column (random signal/fixed effect sizes/total reads) or dataset (fixed signals mean/sd).

	**Simulated small tumours**		**Simulated large tumours**	
	**mean**	**sd**	**mean**	**sd**
Random signal	0	[1.5, 2.5]	0	[4.5, 6.0]
	Applied to all counts; random for each sample row (gRNA) and column (sample).			
Fixed signal for NTC gRNAs	0	[0.5, 1.0]	0	[1.0, 2.0]
	Applied to all 1000 NTC gRNAs; random for each row but fixed across all columns per dataset with effect sizes from [0.9, 1.1] for each column.			
	[-0.3, -0.9]	[1.0, 2.0]	[-1.0, -3.0]	[2.0, 3.0]

Fixed signal for targeting gRNAs	Applied to all 118,461 non-NTC gRNAs; random for each row but fixed across all columns per dataset with effect sizes from [0.9, 1.1] for each column.	
Total number of reads	[14e6, 22e6]	[8e6, 21e6]
	Applied as uniform thinning for each column.	

A total of 100 simulated small tumour and 100 large tumour datasets with 10 samples each were generated. To ascertain the accuracy of each normalisation method tested, we appended the original plasmid counts and the counts of 9 original HCT116/54C cell samples together with 8 simulated small tumours, and three different sets of simulated large tumours (n=10, n=10 and n=8) to generate a combined simulated dataset of 46 columns (samples). Each normalisation method was run for each combined dataset, with normalisation factors calculated from the NTC gRNAs only (apart from the "total count" method) but corrected for total library size. Next, for each simulated sample, the expected $\log_2$ binomial thinning factor for null signals relative to the original plasmid counts (accounting for bootstrap expansion and combined thinning effects) was compared to the calculated $\log_2$ normalisation factor determined by each normalisation method, with the two values expected to be equal. This was performed for a total of 100 random combined datasets to give n=800 (100 dataset × 8 samples) normalisation factor comparisons for simulated small tumours and n=2800 (100 dataset × 28 samples) comparisons for simulated large tumours. As an alternative method for evaluation of normalisation factors, we also compared the normalised size factors (normalised library size relative to comparator sample) to the ratio of summed simulated:comparator counts for gRNAs with null signal in the simulated sample (null signal was defined as the

absolute combined random/fixed signals being < 0.05); both evaluation methods produced similar results so only the results of the first are shown. We also generated 100 simulations of large tumours (n=10) with increased uniform thinning to decrease total counts to [4e6, 5e6], as well as n=100 simulated large tumours with no uniform thinning, which increased total counts to the range [4e7, 6e9]; normalisation factors for these simulated tumours were evaluated in a dataset containing only the 10 simulated columns and the comparator (plasmid) sample.

Estimates of level of negative selection

To estimate the overall level of negative selection present in our tumour samples, we used gRNAs targeting known sets of common essential genes (CEG; n=3891 gRNAs) and non-essential genes (NEG; n=5028 gRNAs) from [6]. As there was a high prevalence of zero counts in the tumour samples, we combined gRNAs into bins of 25 gRNAs, with binning performed separately for CEGs and NEGs and only retaining full bins. The binned gRNAs were further subsampled by 80% to give 124 CEG bins and 161 NEG bins. For each sample, we then calculated the mean $\log_2$ fold-change for CEGs vs NEGs as $\left[mean\left(log_2(CEG\ count)\right) - mean\left(log_2(NEG\ count)\right)\right] - \left[mean\left(log_2(CEG\ count\ in\ inoculum)\right) - mean\left(log_2(NEG\ count\ in\ inoculum)\right)\right]$, where count is the count of a bin and replaced with a pseudo-value of 0.5 if zero (percentage of zeroes was on average <0.2% in large tumours due to binning, and less than 5% in all samples). This value estimates the fold-change depletion of CEGs compared to NEGs over that observed in the inoculum. The estimate was averaged across 100 simulated (binned/subsampled) datasets and all samples in each group (small or large HCT116/54C tumours). To obtain comparable CEG vs NEG $\log_2$ fold-change values for in vitro samples, the process

was repeated for samples of HCT116/54C GeCKO cells, with the second term (inoculum) replaced by the counts of the corresponding cell sample collected at an earlier timepoint. As the periods of culture were different for each cell sample, the average across cell samples was estimated assuming a constant rate of depletion over time by fitting a slope-only linear regression with log2 fold-change for CEGs vs NEGs as the dependent variable and culture period (between collection and the corresponding earlier comparator timepoint) as the explanatory variable.

Supplementary results

Performance of MPM compared to other normalisation methods using simulated data

To investigate the performance of the MPM normalisation method, we performed simulations using binomial thinning. In these simulations, the signal applied is known *a priori* and therefore the expected and actual calculated normalisation factor can be compared as a measure of normalisation performance. We were particularly interested in ensuring the MPM method would function well on data similar to that of the large tumours, which had high count variance, many zero counts and very high read counts for certain gRNAs. We used binomial thinning to generate simulated datasets that contained both simulated large and small tumours, and then compared the calculated normalisation factor to the expected value for the simulated columns.

We first investigated the parameter space for our MPM normalisation method. For simulated small tumours in a combined dataset of small/large tumours, MPM normalisation was generally robust to the pseudocount and *a* clipping parameters, although normalisation factors tended to be biased downwards when *m* trimming was greater than 0, with some interaction with the *a* parameter (Figure S4). For a number of parameter combinations (e.g. a=0, m=0, any pseudocount), the calculated normalisation factors were very close to the expected values (i.e. located close to the line of equality). For simulated large tumours, the parameter with the largest effect was the pseudocount value used. With a high pseudocount (e.g. pseudo=0.5), normalisation factors were biased upwards, that is calculated normalisation factors were greater than expected, indicating that normalised library sizes would be determined to be

larger than they actually were; this property can be seen as the normalisation factors tending to asymptote towards a value determined by the pseudocount. Interestingly, performance using no *a* clipping or *m* trimming (a=0, m=0) was very good with pseudocount of 0.02 or 0.01, with calculated normalisation factors being very similar to expected values. Other combinations of *a* or *m* produced reasonable results with certain pseudocount values, but poorly in others. Overall, taking into account the results for both simulated small and large tumours, no clipping/trimming and a small pseudocount of 0.02 produced very good results. In these simulations, the simulated large tumours had total counts (library sizes) in the range of [8e6, 2.2e7]. With reduced total counts [4e6, 5e6], a smaller pseudocount (0.01) tended to produce better results (Figure S6), while with increased total counts [4e7, 6e9], a larger pseudocount (0.05) tended to produce slightly better results although the influence of the pseudocount was generally reduced in large simulated libraries with fewer zero counts (Figure S6). Nevertheless, a pseudocount of 0.02 generally performed well regardless of library size.

Although we originally expected that clipping/trimming of outlying counts would be beneficial in normalising the high variance/zero-inflated counts of the large tumours, our simulations generally suggested the opposite. A likely explanation is that the use of a log-scale in our MPM calculations greatly reduces the influence of very high counts. A high degree of averaging, first over all valid counts (non-zero in one sample) and then over all pairwise combinations is sufficient to allow reasonable accurate normalisation factors to be calculated despite high count variance. The key determinant in data with many zeroes is that pseudocount used, which can be understood as the unobserved 'average' count where the observed values are zero. A pseudocount of 0.02 worked well in

simulated data with counts in the same range as our data, and was reasonably robust to different library sizes. In addition, the pseudocount only tends to have a noticeable effect in samples with many zeroes, such as simulated large tumours, with the large amount of averaging on a log scale sufficient to dampen its effect in simulated small samples. The use of clipping/trimming, conversely, tends to bias the normalisation factors is various ways, and normalisation is generally better using the simple mean on a log-scale.

We compared MPM (a=0, m=0) normalisation to the commonly used TMM and RLE normalisation methods, as well as the GMPR and the straightforward total count and upper quartile normalisation methods (Figure S7). For TMM and RLE, we also tested the methods with zeroes set to pseudocounts values of 0.5 (commonly used) or 0.02 (preferred MPM parameter) prior to running normalisation. Although the use of pseudocounts with these methods is similar to MPM normalisation, in addition to the differences in averaging methods, MPM differs in that pseudocounts are only used when only one sample has a zero count, which is possible since pairwise comparisons are performed; RLE and TMM do not perform all pairwise comparisons and thus cannot selectively exclude pairwise zeroes from the calculation when pseudocounts are used. For small tumours, MPM and RLE performed well with either a pseudocount of 0.02 or 0.5, as did GMPR (Figure S7A). TMM, regardless of pseudocount used tended to produce upwards biased normalisation factors and the total count and upper quartile performed very poorly. In simulated large tumours, MPM with a pseudocount of 0.02 clearly performed better than all other methods, with most of these producing upward biased normalisation factors in the presence of many zero counts.

Based on these simulations, we chose to use MPM normalisation with no clipping/trimming and a pseudocount of 0.02 in our analyses.

Estimates of level of selection

Based on the depletion of common essential genes compared to non-essential genes, we estimated the log2 fold-change caused by negative selection to be −0.11 in small tumours and −0.39 in large tumours (Figure S8A). This relatively low level of selection, however, may at least partly be due to the relative poor performance of the GeCKOv2 library in negative screens [7] as we estimated the corresponding level of common essential gene depletion to be -0.19 for 40 days of in vitro growth (Figure S8B). Due to the lack of a known set of positive selection genes, we did not estimate levels of positive selection as we reasoned that it would be difficult to distinguish this from the random clonal expansion that we observed.

References

1. Robinson MD, Oshlack A. A scaling normalization method for differential expression analysis of RNA-seq data. Genome Biol. 2010;11:R25.

2. Anders S, Huber W. Differential expression analysis for sequence count data. Genome Biol. 2010;11:R106.

3. Chen L, Reeve J, Zhang L, Huang S, Wang X, Chen J. GMPR: A robust normalization method for zero-inflated count data with application to microbiome sequencing data. PeerJ. 2018;6:e4600.

4. Silverman JD, Roche K, Mukherjee S, David LA. Naught all zeros in sequence count data are the same. Comput Struct Biotechnol J. 2020;18:2789–98.

5. Gerard D. Data-based RNA-seq simulations by binomial thinning. BMC Bioinformatics. 2020;21:206.

6. Kim E, Hart T. Improved analysis of CRISPR fitness screens and reduced off-target effects with the BAGEL2 gene essentiality classifier. Genome Med. 2021;13:2.

7. Doench JG, Fusi N, Sullender M, Hegde M, Vaimberg EW, Donovan KF, Smith I, Tothova Z, Wilen C, Orchard R, Virgin HW, Listgarten J, Root DE. Optimized sgRNA design to maximize activity and minimize off-target effects of CRISPR-Cas9. Nat Biotechnol. 2016;34:184–91.
